# Double Stokes polarimetric microscopy for chiral fibrillar aggregates

**DOI:** 10.1038/s41598-025-86893-0

**Published:** 2025-02-06

**Authors:** Viktoras Mazeika, Kamdin Mirsanaye, Leonardo Uribe Castaño, Serguei Krouglov, Mehdi Alizadeh, Mykolas Maciulis, Lukas Kontenis, Vitalijus Karabanovas, Virginijus Barzda

**Affiliations:** 1 Institute of Biosciences, Life Sciences Center, Vilnius University, Vilnius, Lithuania; 2 Laser Research Centre, Faculty of Physics, Vilnius University, Vilnius, Lithuania; 3 Department of Chemical and Physical Sciences, University of Toronto Mississauga, Mississauga, ON Canada; 4 Department of Physics, University of Toronto, Toronto, ON Canada; 5 Wellman Center for Photomedicine and Center for Systems Biology, Massachusetts General Hospital and Harvard Medical School, Boston, MA USA; 6 Light Conversion, Vilnius, Lithuania; 7 Biomedical Physics Laboratory, National Cancer Institute, Vilnius, Lithuania

**Keywords:** Multiphoton microscopy, Polarization microscopy, Biological physics, Nonlinear optics

## Abstract

Second harmonic generation (SHG) microscopy is a powerful tool for imaging collagen and other noncentrosymmetric fibrillar structures in biological tissue. Polarimetric SHG measurements provide ultrastructural information about the fibrillar organization in a focal volume (voxel). We present a reduced nonlinear polarimetry method named double Stokes polarimetry (DSP) for quick characterization of chiral $C_6$ symmetry fibers without data fitting that simplifies and speeds up the polarimetric analysis. The method is based on double Stokes-Mueller polarimetry and uses linear and circular incident and outgoing polarization states. The analytical expressions of DSP polarimetric parameters are defined in terms of conventional SHG Stokes vector components. A complex chiral susceptibility (CCS) model is assumed to derive expressions of ultrastructural parameters consisting of the magnitude and phase of molecular complex-valued chiral susceptibility ratio, real-valued achiral ratio, and fiber orientation in a voxel. The ultrastructural parameters are expressed in terms of directly measurable DSP polarimetric parameters. DSP is validated with rat tail tendons sectioned at different orientations. DSP can be applied to investigate the origin of chiral complex-valued susceptibility of collagen, to study modifications of collagen in cancerous tissue, and to map ultrastructural parameters of large areas for whole-slide histopathology.

## Introduction

Second harmonic generation (SHG) microscopy is a label-free visualization technique for non-centrosymmetric fibrillar structures found in biological organisms^1–4^. SHG intensity depends on the incident and outgoing polarizations and on the sample structure. Therefore, polarimetric SHG microscopy can be applied to obtain ultrastructural information from each focal volume (voxel) of the imaged sample^5,6^.

Several nonlinear polarimetry methods have been introduced to investigate fibrillar biological structures. Complete 2-dimensional polarimetric characterization of the samples is obtained with double Stokes-Mueller polarimetry (DSMP)^7^. Reduced polarimetry techniques can use linear incident^8–11^and outgoing^5,12–16^polarization states for polarimetric measurements with subsequent nonlinear fitting of the polarimetric data to extract the ultrastructural parameters. The linear polarization-in polarization-out (PIPO) method^5,16^allows one to determine the achiral and chiral molecular susceptibility ratios^12,17^. To speed up the extraction of ultrastructural parameters, a Fourier transform based algorithm^11^and phasor approach^18^can be employed for incident polarization variation measurements. Circular polarization states have also been used to extract the susceptibility ratios from orientation-independent measurements^12,19,20^.

Polarimetric measurements can be described by Jones or Stokes formalism. Stokes formalism uses signal intensities, which can be measured experimentally. The extent of depolarization can be estimated via degree of polarization (DOP). Therefore, it is convenient to use Stokes formalism for describing polarimetric data. For fast detection of Stokes parameters, a four-channel Stokes polarimeter has been demonstrated to simultaneously obtain the whole SHG Stokes vector and to determine the degree of polarization and anisotropy values, as well as to assess the material birefringence^21,22^.

Most polarimetry techniques require data fitting or Fourier analysis on a pixel-by-pixel basis to extract the ultrastructural parameters from the measured images. In this paper, we introduce a reduced polarimetry technique called double Stokes polarimetry (DSP), which allows for quick calculation of the ultrastructural parameters directly from the polarimetry images by using analytical expressions derived from the DSMP formalism. DSP resembles conventional Stokes polarimetry. Normalized differences and sums of SHG Stokes vector components are combined to calculate the polarimetric parameters, including, SHG circular dichroism ($SHG_{CD}$)^23^, SHG linear dichroism ($SHG_{LD}$)^24,25^, and circular anisotropy of circular dichroism ($CA_{CD}$)^26^. $SHG_{CD}$ and $CA_{CD}$ have been used for structural investigations of tissue samples^24,27,28^. DSP polarimetric parameters were employed for multiparametric analysis with machine learning for cancer diagnostics^24,29^. We present new DSP polarimetric parameters that are used to derive the expressions of the ultrastructural parameters. The expressions utilize linear and circular incident and outgoing polarization states. The polarimetric and ultrastructural parameters are verified experimentally using rat tail tendon (RTT) samples cut at different angles with respect to the fiber axis. The analytical expressions of DSP parameters can be used for fast calculation of ultrastructural parameters without fitting, which speeds up data processing. DSP polarimetry is relevant for large-area imaging, with applications in nonlinear digital histopathology.

## Theoretical considerations

The SHG Stokes vector, *s*, components can be obtained from polarimetric SHG intensity measurements involving outgoing horizontally (HLP), vertically (VLP), $45^{\circ }$ (45) and $-45^{\circ }$ (−45) diagonally linearly polarized states, and right-handed (RCP) and left-handed (LCP) circularly polarized states:

1 $$\begin{aligned} s = \begin{bmatrix} s^m_0\\ s^m_1\\ s^m_2\\ s^m_3\\ \end{bmatrix} = \begin{bmatrix} I^m_{HLP}+I^m_{VLP}\\ I^m_{HLP}-I^m_{VLP}\\ I^m_{45}-I^m_{-45}\\ I^m_{RCP}-I^m_{LCP}\\ \end{bmatrix}. \end{aligned}$$

The $s_0$ Stokes vector component shows the total signal intensity. If SHG is partially polarized, then $s_0^2\ge s_1^2+s_2^2+s_3^2.$ DSP formalism assumes that the fundamental radiation and SHG are fully polarized^7,30^. Therefore, the $s_0$ component of SHG is calculated from the other Stokes components according to $s_0^2=s_1^2+s_2^2+s_3^2$ and applied to filter the polarimetric measurements. The effect of depolarization on the polarimetric and ultrastructural parameters is tested experimentally. The comparison shows similar distributions of experimentally obtained polarimetric and ultrastructural parameters using unfiltered and filtered $s_0$ component (see Supplementary Material G). Therefore, the assumption of pure states is valid and will be applied in the following sections. The SHG Stokes vector is obtained for a particular incident polarization state, which is indicated by the superscript index *m* in Eq. (1). Incident polarization states can be described by the double Stokes vector^7^, *S*, as outlined in Supplementary Material A. Only HLP, VLP, 45, $-45$, RCP and LCP states are used in this study.

The nonlinear optical properties of the material are described by the double Mueller matrix *M *from the double Stokes-Mueller equation^7^:

2 $$\begin{aligned} s = MS \end{aligned}$$

The laboratory reference frame nonlinear susceptibilities can be calculated from the Mueller matrix^7^ (see Supplementary Material B, C). By assuming a structural symmetry model and using tensor rotation, the molecular susceptibility tensor components can be calculated from the laboratory reference frame susceptibility tensor (see Supplementary Material B). Biological structures such as collagen and myosin can be modeled as chiral fibers with $C_6$ symmetry^17^. In this case, the molecular susceptibility tensor contains only 4 independent components which can be expressed via three ratios: $\frac{\chi _{zzz}}{\chi _{zxx}}$, $\frac{\chi _{xyz}}{\chi _{zxx}}$, $\frac{\chi _{xxz}}{\chi _{zxx}}$, with the fiber axis oriented along the *z* direction of the molecular reference frame. The relations between laboratory and molecular frame susceptibilities assume dipole approximation and contain the fiber in-image-plane orientation angle $\delta$ and out-of-image-plane tilt angle $\alpha$ (see Supplementary Material D). Since only the projection of the fiber onto the image plane can be directly measured, the laboratory reference frame susceptibilities are expressed via effective achiral (*R*) and chiral (*C*) molecular susceptibility ratios implicitly containing $\alpha$ as follows^12^:

3 $$\begin{aligned} & R=\frac{\chi _{zzz}}{\chi _{zxx}}\cos ^2\alpha +\left( 1+2\frac{\chi _{xxz}}{\chi _{zxx}}\right) \sin ^2\alpha , \end{aligned}$$

4 $$\begin{aligned} & C=\frac{\left| \chi _{xyz}\right| }{\chi _{zxx}}\sin \alpha ,~~~~~~~ \chi _{xyz}=\left| \chi _{xyz}\right| e^{i\Delta }, \end{aligned}$$

5 $$\begin{aligned} & \chi _{zxx}\simeq \chi _{xxz}. \end{aligned}$$

**Fig. 1:**
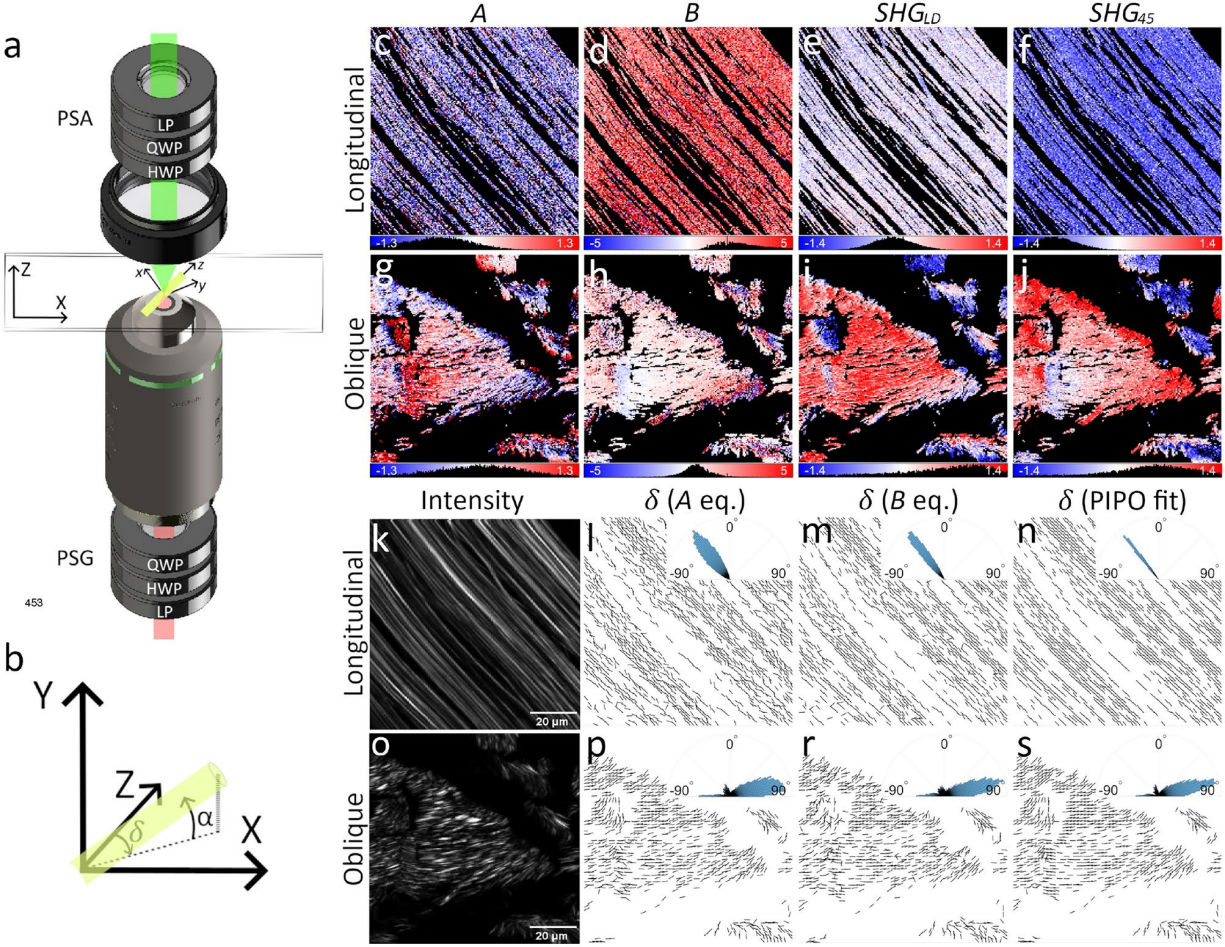
DSP experimental setup and RTT sample maps of the orientation $\delta$-dependent parameters. The scheme of the DSP measurement is shown in (**a**) (PSG - polarimetric state generator, PSA - polarimetric state analyzer, LP - linear polarizer, HWP - half-wave plate, QWP - quarter-wave plate). The laboratory reference frame XYZ is presented in (**a**, **b**) and the molecular reference frame xyz is shown in (**a**) together with the fiber. A collagen fiber is shown in the laboratory frame coordinate system (**b**) with indicated $\alpha$ and $\delta$ angles. The maps of the $\delta$-dependent polarimetric parameters *A* (**c**, **g**), *B* (**d**, **h**), $SHG_{LD}$ (**e**, **i**), and $SHG_{45}$ (**f**, **j**) are shown for the longitudinal (**c**-**f**) and oblique cuts (**g**-**j**). The $\delta$ orientation maps calculated from *A* (**l**, **p**), *B* (**m**, **r**) and obtained from the PIPO fits (**n**, **s**) are shown for the longitudinal (**l**-**n**) and oblique (**p**-**s**) cuts. For reference, the polarization-independent SHG intensity images are obtained for the longitudinal (**k**) and oblique (**o**) cuts by summing all 36 polarization measurements used for DSP.

The complex chiral susceptibility (CCS) model is assumed where achiral ratios are real and the chiral ratio $\chi _{xyz}$ is complex-valued, as noted by Eq. (4). Therefore, *C* represents the ratio magnitude multiplied by $\sin {\alpha }$. The phase $\Delta$ represents the retardance between the achiral and chiral molecular susceptibility components^7^. The out-of-plane tilt $\alpha$ is the angle between the molecular *z* axis and its projection onto the image plane. The in-plane orientation angle $\delta$ is defined as the angle between the projection of the molecular *z* axis onto the image plane and the *Z* axis of the laboratory reference frame. The image plane is defined in the laboratory reference frame by the *XZ* axes and the *Y* axis is oriented along the direction of light propagation (see Fig. 1 a, b).

### DSP polarimetric parameters

DSP polarimetric parameters are based on the ratios of sums and differences of SHG Stokes vector components obtained with orthogonal incident laser polarizations. The expressions of the sums and differences for incident HLP, VLP, 45, $-45$, RCP and LCP states are outlined in Supplementary Material E and F. These expressions are given in terms of the molecular susceptibility ratios and $\delta$, and can be used to derive the DSP polarimetric parameters. The polarimetric parameters, which depend on the fiber orientation, can be used for the calculation of $\delta$, while $\delta$-independent parameters can be used to calculate the *R* ratio, *C* magnitude and phase difference $\Delta$. The polarimetric parameters are summarized in Table 1.

**Table 1:** List of DSP polarimetric parameters calculated using CCS model and their biophysical significance.

Parameter	Name/ description	Incident polarization	Outgoing polarization	Biophysical significance	References
$SHG_{LD}$	Linear dichroism	Linear	Total int.	Depends on fiber orientation ($2\delta$).	^24,25,29^
$SHG_{45}$	*Diagonal dichroism	*Diagonal	Total int.	Depends on fiber orientation ($2\delta$).	-
*A*	Difference over the sum of linear and diagonal dichroism	Linear and diagonal	Total int.	Proxy for calculating $\delta$, depends on $\sigma$.	-
*B*	Ratio of $s_2$ and $s_1$ of SHG for circular incident states	Circular	Linear and diagonal	Proxy for calculating $\delta$, depends on $\sigma$.	-
$CA_{CD}$	Circular anisotropy of circular dichroism	Circular	Circular	Independent of $\delta$, used to calculate R ratio.	^25,26^
*L*	Normalized sum of linear anisotropy of linear dichroism and diagonal anisotropy of diagonal dichroism	Linear and diagonal	Linear and diagonal	Independent of $\delta$, depends on R ratio.	^37^
*T*	Normalized difference of diagonal anisotropy of linear dichroism and linear anisotropy of diagonal dichroism	Linear and diagonal	Linear and diagonal	Independent of $\delta$, depends on *R* and $C\cos {\Delta }$, reveals fiber polarity.	^37^
*H*	Ratio of *T* and *L* parameters	Linear and diagonal	Linear and diagonal	Independent of $\delta$, depends on *R* and $C\cos {\Delta }$, reveals fiber polarity, direct measure of $3\sigma$.	-
$SHG_{CD}$	Circular dichroism	Circular	Total int.	Independent of $\delta$, depends on *R* and $C\sin {\Delta }$ (fiber polarity and retardance). $SHG_{CD}\approx 0$ for achiral and in-plane fibers.	^24,25,27,29,38^
*W*	Normalized sum of all incident linear and diagonal $s_3$ of SHG	Linear and diagonal	Circular	Independent of $\delta$, depends on *R* and $C\sin {\Delta }$ (fiber polarity and retardance). $SHG_{CD}\approx 0$ for achiral and in-plane fibers.	-
*D*	Phase difference between achiral and chiral susceptibility components	Linear and diagonal	Linear, diagonal and circular	Calculated from $-W/T$, direct measure of $\tan {\Delta }$ for tilted chiral fibers.	-

*Diagonal designates the use of 45 and $-45$ linear states.

#### Polarimetric parameters dependent on $\delta$

The *A* and *B* polarimetric parameters can be employed for the calculation of $\delta$. The *A* parameter is obtained by measuring $s_0$ for incident horizontal, vertical, and diagonal linear polarizations:

6 $$\begin{aligned} A = \frac{s_0^{HLP}-s_0^{VLP}-(s_0^{+45}-s_0^{-45})}{s_0^{HLP}-s_0^{VLP}+(s_0^{+45}-s_0^{-45})} = \frac{-(a+b)\cos (2\delta )-(a-b)\sin (2\delta )}{-(a-b)\cos (2\delta )+(a+b)\sin (2\delta )}\approx \frac{-(1+\sigma )\cos (2\delta )-(1-\sigma )\sin (2\delta )}{-(1-\sigma )\cos (2\delta )+(1+\sigma )\sin (2\delta )}, \end{aligned}$$

where $a=(R^2-1+4C^2)$ and $b=2(R-1)C\cos \Delta$, and

7 $$\begin{aligned} \sigma =\frac{2C\cos \Delta }{R+1}. \end{aligned}$$

The approximate *A* equation is expressed in terms of $\sigma$ by assuming $|R^2-1|>>4C^2$. Circular incident and linear outgoing polarization states can be used to calculate the *B* parameter:

8 $$\begin{aligned} B = \frac{s_2^{RCP}+s_2^{LCP}}{s_1^{RCP}+s_1^{LCP}}= \frac{-\sin \left( 2\delta \right) +2\sigma \cos \left( 2\delta \right) }{\cos \left( 2\delta \right) +2\sigma \sin \left( 2\delta \right) }. \end{aligned}$$

Both *A* and *B* depend on the $$\sigma$$ parameter, which appears in all equations involving linear polarization states. It is an important parameter that characterizes the relative magnitude between $$C\cos {\Delta }$$ and *R* ratio. The $$\sigma$$ allows for correction of the shift in the orientation-dependent polarimetric parameters due to the fiber chirality. The $$\delta$$ is uniquely determined from *A* and *B* parameters in [$$-\frac{\pi }{4};\frac{\pi }{4}$$] range, which can be extended to [$$-\frac{\pi }{2};\frac{\pi }{2}$$] by using the signs of $$SHG_{LD}$$ and SHG diagonal linear dichroism ($$SHG_{45})$$:

9 $$\begin{aligned} & SHG_{LD}=2\frac{(s_0^{HLP}-s_0^{VLP})}{(s_0^{HLP}+s_0^{VLP})}\approx -8\frac{\frac{(R-1)}{(R-3)}\sqrt{1+\sigma ^2}\cos \left( 2\delta +2\tan ^{-1}\frac{\sigma }{1+\sqrt{1+\sigma ^2}}\right) }{\sqrt{1+(2\sigma )^2}\cos \left( 4\delta +2\tan ^{-1}\frac{2\sigma }{1+\sqrt{1+(2\sigma )^2}}\right) +\kappa }, \end{aligned}$$

10 $$\begin{aligned} & SHG_{45}=2\frac{(s_0^{+45}-s_0^{-45})}{(s_0^{+45}+s_0^{-45})}\approx 8\frac{\frac{(R-1)}{(R-3)}\sqrt{1+\sigma ^2}\cos \left( 2\delta -2\tan ^{-1}\frac{1}{\sigma +\sqrt{\sigma ^2+1}}\right) }{-\sqrt{1+(2\sigma )^2}\cos \left( 4\delta +2\tan ^{-1}\frac{2\sigma }{1+\sqrt{1+(2\sigma )^2}}\right) +\kappa }, \end{aligned}$$

11 $$\begin{aligned} & \kappa =\frac{(3R^2+2R+7)+16C^2}{(R+1)(R-3)}. \end{aligned}$$

Note that both $SHG_{LD}$ and $SHG_{45}$ are modulated by $2\delta$, with the phase shift dependent on $\sigma$.

**Table 2:** List of ultrastructural parameters obtained from DSP polarimetric parameters and their biophysical significance.

Parameter	Name/ description	Related polarimetric parameters	Biophysical significance	References
$\delta$	In-image-plane fiber orientation angle	*A*, *B*, $SHG_{LD}$, $SHG_{45}$	Fiber in-image-plane orientation	^5^
*R*	Achiral susceptibility ratio	$CA_{CD}$, *L*	Reflects the orientation distribution of nonlinear dipoles with respect to the fiber axis	^5^
*C*	Magnitude of chiral susceptibility ratio	*H*, *T*, $SHG_{CD}$, *W*	Reflects the helical arrangement of dipoles with respect to the fiber axis	^35^
$\Delta$	Phase difference between achiral and chiral susceptibility ratios	*H*, *T*, $SHG_{CD}$, *W*, *D*	Phase difference between achiral and chiral nonlinear susceptibility tensor component ratios	^17^

#### Polarimetric parameters independent of $\delta$

Some linear polarization states can be combined, or combinations of circular and linear polarizations can be used to obtain $\delta$-independent polarimetric parameters. These parameters are employed to calculate *R*, *C* and $\Delta$.

The polarimetric parameters dependent on *R* and independent of $\Delta$ are obtained by using only linear (*L* parameter) or only circular ($CA_{CD}$ parameter) polarization states:

12 $$\begin{aligned} & L = \frac{s_1^{HLP}-s_1^{VLP}+s_2^{+45}-s_2^{-45}}{s_0^{HLP}+s_0^{VLP}+s_0^{+45}+s_0^{-45}} = \frac{2(R+1)^2-16C^2}{3R^2+2R+7+16C^2}, \end{aligned}$$

13 $$\begin{aligned} & CA_{CD} = 2\frac{s_3^{RCP}-s_3^{LCP}}{s_0^{RCP}+s_0^{LCP}} = \frac{8[(R-1)+2C^2]}{(R-1)^2+8C^2+4}. \end{aligned}$$

Linear polarization states measured with a $45^{\circ }$ angle between the polarizer and analyzer provide the parameters dependent on $C\cos {\Delta }$:

14 $$\begin{aligned} & T= \frac{s_2^{HLP}-s_2^{VLP}-(s_1^{+45}-s_1^{-45})}{s_0^{HLP}+s_0^{VLP}+s_0^{+45}+s_0^{-45}} = \frac{12C\cos \Delta (R+1)}{3R^2+2R+7+16C^2}, \end{aligned}$$

15 $$\begin{aligned} & H = \frac{T}{L} = \frac{s_2^{HLP}-s_2^{VLP}-(s_1^{+45}-s_1^{-45})}{s_1^{HLP}-s_1^{VLP}+s_2^{+45}-s_2^{-45}} = \frac{6C\cos \Delta (R+1)}{(R+1)^2-8C^2}\approx \frac{6C\cos \Delta }{(R+1)} = 3\sigma . \end{aligned}$$

*H* is the ratio of *T* and *L* parameters. $(R+1)^2>>8C^2$ is assumed for the approximate calculation of $\sigma$ from the *H* parameter. *H* can be used to obtain $\sigma$ values directly from the experiment.

The polarimetric parameters dependent on $C\sin {\Delta }$ can be obtained by employing circular incident polarization states, or linear incident and circular outgoing polarization states:

16 $$\begin{aligned} & SHG_{CD} = 2\frac{s_0^{RCP}-s_0^{LCP}}{s_0^{RCP}+s_0^{LCP}} = \frac{-8C\sin {\Delta }(R+1)}{(R-1)^2+8C^2+4}, \end{aligned}$$

17 $$\begin{aligned} & W = \frac{s_3^{HLP}+s_3^{VLP}+s_3^{+45}+s_3^{-45}}{s_0^{HLP}+s_0^{VLP}+s_0^{+45}+s_0^{-45}} = \frac{-12C\sin \Delta (R+1)}{3R^2+2R+7+16C^2}. \end{aligned}$$

When $\Delta$ and/or *C* are equal to 0, $SHG_{CD}$ and *W* values are 0, as in the case with achiral fibers or fibers oriented in the image plane.

### Ultrastructural parameters calculated from DSP polarimetric parameters

Five ultrastructural parameters can be used to characterize chiral fibrillar structures: the achiral susceptibility ratio *R*, the magnitude of chiral susceptibility ratio *C*, the retardance between the achiral and chiral susceptibility components $\Delta$, fiber in-image-plane orientation angle $\delta$, and fiber out-of-image-plane tilt angle $\alpha$. All ultrastructural parameters except $\alpha$ can be calculated from various combinations of DSP polarimetric parameters. The $\alpha$ parameter can be obtained by assuming characteristic molecular susceptibility ratios of the fiber under consideration^16^. The ultrastructural parameters are summarized in Table 2.

#### In-image-plane fiber orientation angle $\delta$

The $\delta$ angle can be calculated using the *A* parameter (Eq. (6)) which combines linear polarization states, or the *B* parameter (Eq. (8)) which uses circular incident and linear outgoing polarization states:

18 $$\begin{aligned} & \tan 2\delta \approx \frac{(A-1)-(A+1)\sigma }{(A+1)+(A-1)\sigma }, \end{aligned}$$

19 $$\begin{aligned} & \tan 2\delta =\frac{-B+2\sigma }{1+2\sigma B}. \end{aligned}$$

The equations contain $\sigma$, which can be determined from the *H* parameter (Eq. (15)). Both equations are valid with any *C* ratio value. The $\delta$ values can be uniquely determined in [$-\frac{\pi }{4};\frac{\pi }{4}$] range, which can be extended to [$-\frac{\pi }{2};\frac{\pi }{2}$] by using the sign dependence of $SHG_{LD}$ and $SHG_{45}$.

#### Achiral susceptibility ratio R

The *R* ratio can be calculated using linear, circular, or a combination of linear and circular polarization states. For circular polarization states, $CA_{CD}$ can be used, with the assumption that $C^2$is small^24,29^:

20 $$\begin{aligned} R = 1+\frac{4}{CA_{CD}}\pm 2\sqrt{\frac{4}{(CA_{CD})^2}-1-2C^2+\frac{4C^2}{CA_{CD}}}\approx 1+\frac{4}{CA_{CD}}\pm 2\sqrt{\frac{4}{(CA_{CD})^2}-1}. \end{aligned}$$

The assumption of small $C^2$ is not needed if both $CA_{CD}$ and *L* parameters are employed, but requires the use of both linear and circular polarization states:

21 $$\begin{aligned} R=\frac{\pm 2\sqrt{3{\left( CA_{CD} -2\right) }{\left( L -2\right) }{\left( 2CA_{CD} +6L +CA_{CD} L +4\right) }} + 6L +3CA_{CD} L +12}{4CA_{CD} +6L -CA_{CD} L -4}. \end{aligned}$$

#### Chiral susceptibility ratio C and retardance $\Delta$

The magnitude of the chiral susceptibility ratio *C* and the retardance $\Delta$ can be separated by obtaining $C\cos {\Delta }$ from the measurements with linear polarizations (*T* or *H* parameters) and $C\sin {\Delta }$ from the measurements with circular polarizations ($SHG_{CD}$ or *W* parameters):

22 $$\begin{aligned} & C\cos \Delta = \frac{T(3R^2+2R+7+16C^2)}{12(R+1)}, \end{aligned}$$

23 $$\begin{aligned} & C\cos \Delta = \frac{H[(R+1)^2-8C^2]}{6(R+1)}, \end{aligned}$$

24 $$\begin{aligned} & C\sin \Delta =- \frac{SHG_{CD}((R-1)^2+8C^2+4)}{8(R+1)}, \end{aligned}$$

25 $$\begin{aligned} & C\sin \Delta =- \frac{W(3R^2+2R+7+16C^2)}{12(R+1)}. \end{aligned}$$

The magnitude *C* can be calculated from Eqs. (23) and (24), or, alternatively, from Eqs. (22) and (25) by $\sqrt{(C\cos {\Delta })^2+(C\sin {\Delta })^2}$ and determining the sign from $C\cos {\Delta }$.

By taking the expressions of $C\sin \Delta$ and $C\cos \Delta$, $\tan \Delta$ can be calculated:

26 $$\begin{aligned} \tan \Delta =-\frac{3}{4}\frac{SHG_{CD}((R-1)^2+8C^2+4)}{H((R+1)^2-8C^2)}\approx -\frac{3}{4}\frac{SHG_{CD}((R-1)^2+4)}{H(R+1)^2}. \end{aligned}$$

The approximation $(R+1)^2>>8C^2$ is assumed. Alternatively, $\tan \Delta$ can be deduced directly from SHG Stokes vector components as the parameter *D*:

27 $$\begin{aligned} D=-\frac{W}{T}=\tan \Delta =-\frac{(s_3^{HLP}+s_3^{VLP})+(s_3^{+45}+s_3^{-45})}{(s_2^{HLP}-s_2^{VLP})-(s_1^{+45}-s_1^{-45})}. \end{aligned}$$

Nonzero $\Delta$ values signify the presence of imaginary chiral second-order susceptibility. Note that due to $C\sin {\Delta }/C\cos {\Delta }$, Eqs. (26) and (27) do not accurately determine $\Delta$ when $C\approx 0$ for achiral fibers or fibers in the image plane. To mitigate this, pixels with $C\approx 0$ can be removed from $\Delta$ maps. Small values of *H*, *T*, $SHG_{CD}$ and *W* parameters simultaneously indicate that *C* values are small. $\Delta$ has been examined with full DSMP measurements^17^, and can also be assessed indirectly with $SHG_{CD}$ measurements^27^. On the other hand, Eq. (27) provides a direct measurement of $\Delta$ by using linear incident polarization states and measuring all three outgoing Stokes vector components.

## Results and discussion

The expressions of polarimetric and ultrastructural parameters (Tables 1 and 2, respectively) are experimentally validated by imaging RTT sectioned longitudinally and obliquely with respect to the fiber axis (Fig. 1 k, o). The parameters are calculated without fitting, from SHG Stokes vector components obtained from the polarimetric intensity images. Therefore, DSP is applicable for fast analysis of large-area high-resolution images. The pure states assumption is employed by calculating $s_0^2=s_1^2+s_2^2+s_3^2$ from the experimental data. For comparison, the polarimetric and ultrastructural parameters are obtained using directly measured $s_0$, without filtering for the pure states. The distributions of unfiltered and filtered parameters are presented in Supplementary Material G, showing that filtering for pure polarization states has negligible influence on the parameters. The DOP for both samples (longitudinal and oblique cuts) was around 0.9.

Fig. 1 a shows the scheme of the measurement. The incident polarization of the laser beam is set with the polarization state generator (PSG) and the polarization of the outgoing SHG is probed with the polarization state analyzer (PSA). HLP, VLP, 45, $-45$, RCP, and LCP states are used for the measurements. Maps of four ultrastructural parameters are obtained from the DSP polarimetric parameters and are compared with the fitting results from well-established PIPO measurements^5,12,16^.

### Fiber in-plane orientation $\delta$

The fiber orientation $\delta$ is obtained by employing *A*, *B*, $SHG_{LD}$ and $SHG_{45}$ polarimetric parameters (Eqs. (6), (8), (9) and (10), Fig. 1 c-j). The parameter values are uniformly distributed in the longitudinal cut tendon due to fibers aligned in the same direction (Fig. 1 c-f). In the oblique cut sample, the fibers are oriented in different directions, which is reflected by different values in the maps (Fig. 1 g-j). Note that due to the periodicity of the functions describing the polarimetric parameters, the value correspondence with $\delta$ is not unique. In addition, the modulation of each polarimetric parameter slightly deviates from the actual fiber orientation for larger $\sigma$ values. Therefore, $\delta$ can be directly estimated from individual *A*, *B*, $SHG_{LD}$ and $SHG_{45}$ maps only for relatively narrow variation of fiber orientations ($<90^{\circ }$ spread) and with fibers oriented in the image plane.

A combination of several polarimetric parameters can be used to obtain $\delta$ with high precision by correcting for the shift in orientation modulation due to fiber chirality. Two alternative calculations are presented based on incident linear (*A* parameter), or incident circular and outgoing linear (*B* parameter) polarization states. Both equations contain the $\sigma$ parameter, which affects $\delta$ calculation results for tilted fibers. The $\sigma$ can be directly measured from the *H* parameter using linear incident and outgoing polarization states (Eq. (15), Fig. 3 b, f). The fiber orientation maps obtained from *A* (Eq. (18)) and *B* (Eq. (19)) parameters are shown in Fig. 1 l, p, and m, r, respectively. For comparison, $\delta$ maps obtained from PIPO fits are shown in Fig. 1 n, s. All three methods give similar orientation maps (median and interquartile range (IQR) values): $-39^{\circ }\pm 18^{\circ }$ (Eq. (19), A eq.), $-39^{\circ }\pm 10^{\circ }$ (Eq. (18), B eq.) and $-38^{\circ }\pm 4^{\circ }$ (PIPO fit) for the longitudinal cut sample, $58^{\circ }\pm 91^{\circ }$ (Eq. (19), A eq.), $56^{\circ }\pm 93^{\circ }$ (Eq. (18), B eq.) and $58^{\circ }\pm 87^{\circ }$ (PIPO fit) for the oblique cut sample, though the $\delta$ distribution of the PIPO fit is narrower (compare the insets in Fig. 1 l, m with n) due to fitting that reduces the influence of data noise. The orientation maps match well the fiber orientations visible in the intensity images (Fig. 1 k, o). Note that $\delta$ is uniquely determined with *A* or *B* parameters in the [$-\frac{\pi }{4};\frac{\pi }{4}$] range, which can be extended to [$-\frac{\pi }{2};\frac{\pi }{2}$] by taking into account the signs of $SHG_{LD}$ and $SHG_{45}$. Overall, the *B* parameter gives more accurate $\delta$ values than *A* (compare insets in Fig. 1 l, m), but it requires to measure circular and linear polarization states, while *A* employs only linear polarization states. The direct use of $s_0$ without filtering for pure states does not significantly affect the distributions of *A*, *B* and $\delta$ values as shown in Supplementary Material G. Algebraic calculation of $\delta$ images from the polarimetric parameters takes several seconds, while PIPO fitting requires tens of minutes. The fast calculation of $\delta$ is important for large-area imaging.

**Fig. 2:**
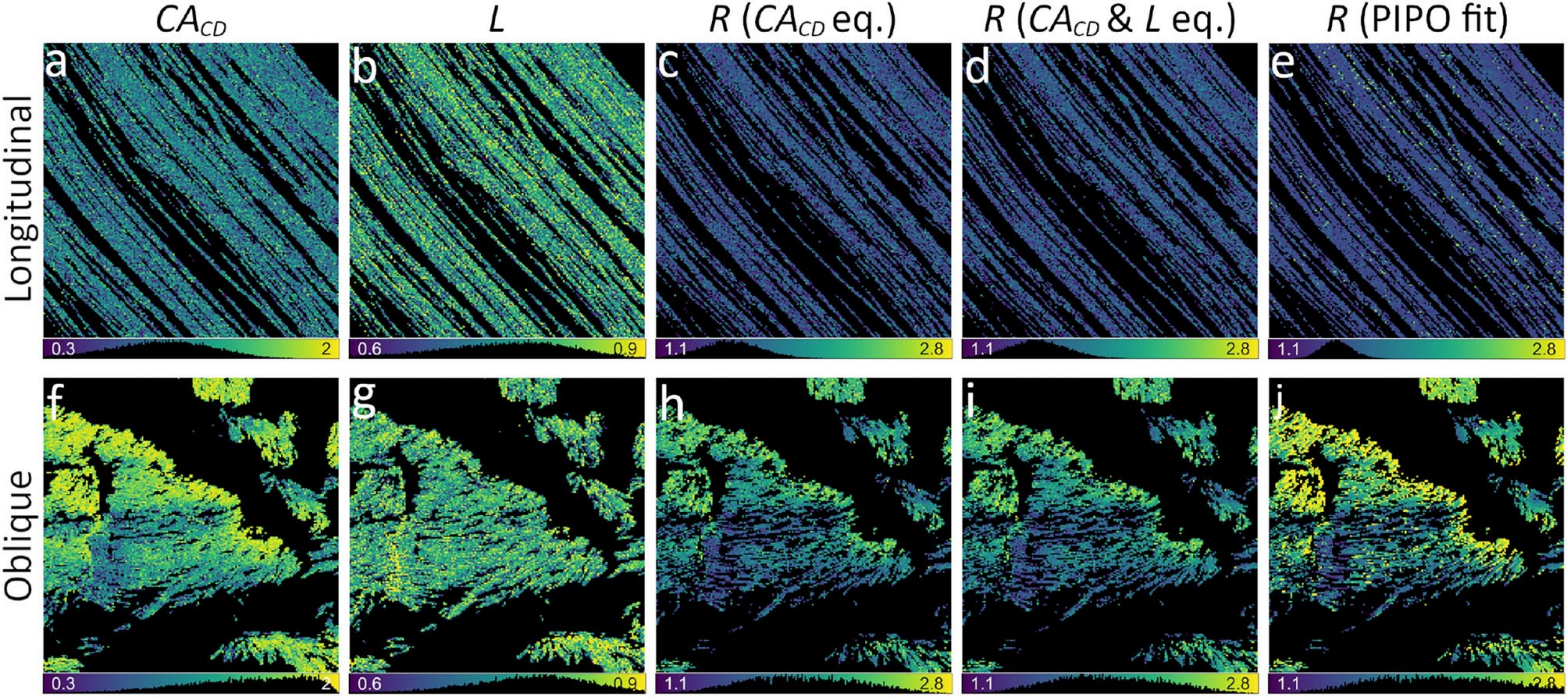
Maps of $CA_{CD}$ (**a**, **f**) and *L* (**b**, **g**) polarimetric parameters and *R* ratio maps calculated with $CA_{CD}$ Eq. (20) (**c**, **h**), $CA_{CD} \& L$ Eq. (21) (**d**, **i**), and PIPO fitting (**e**, **j**) for the longitudinal (**a**-**e**) and oblique (**f**-**j**) cut RTT. Parameter histograms, together with lower and upper bounds, are shown under the corresponding maps. The color lookup bars are combined with the histograms. The same scan areas are analyzed as in Fig. 1.

**Fig. 3:**
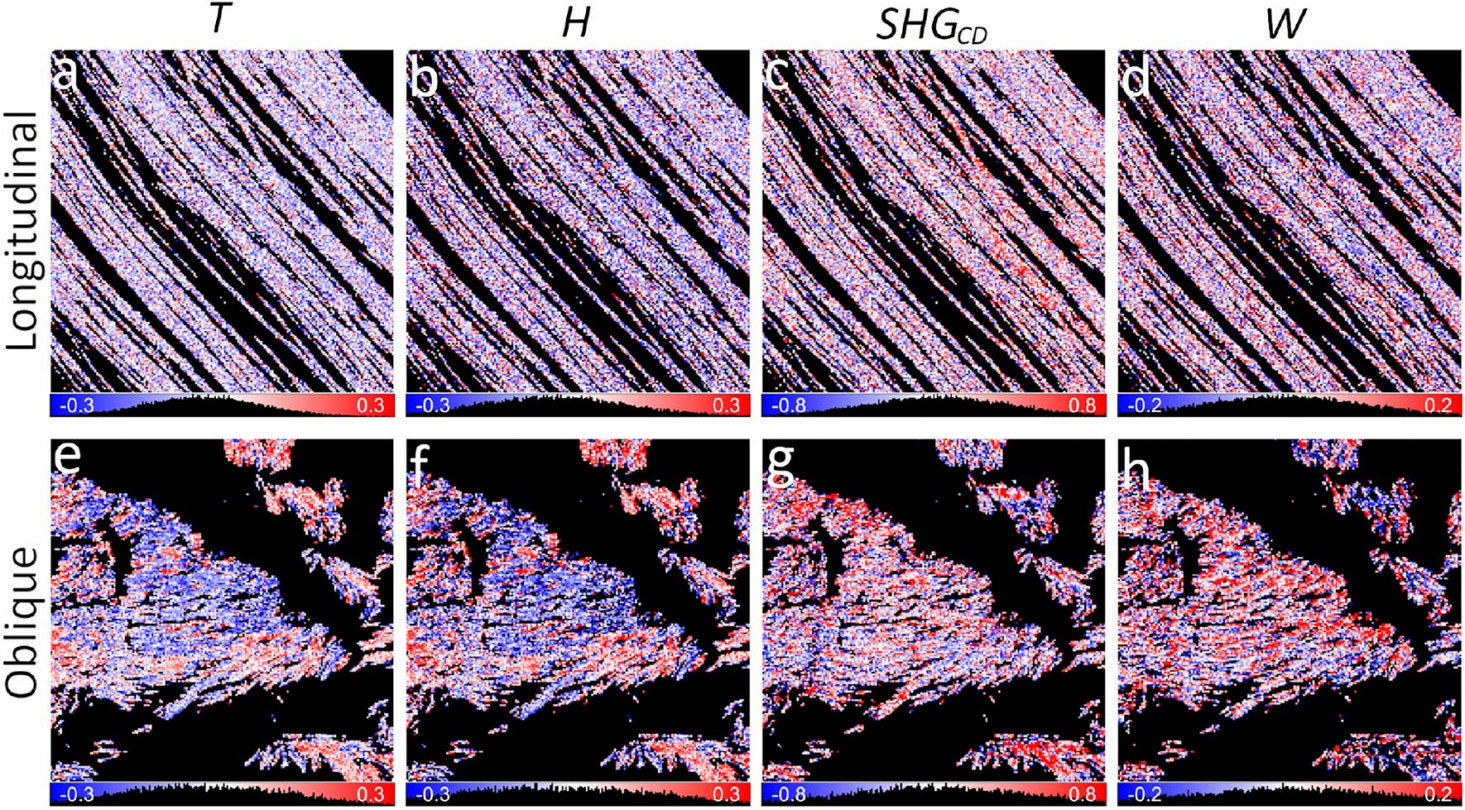
Maps of *T* (**a**, **e**), *H* (**b**, **f**), $SHG_{CD}$ (**c**, **g**) and *W* (**d**, **h**) polarimetric parameters of the longitudinal **(a**-**d**) and oblique (**e**-**h**) cuts of RTT. Parameter histograms are shown under the corresponding maps. The color lookup bars are combined with the histograms. The same scan areas are shown as in Figs. 1, 2.

### *R* ratio

The *R* ratio can be obtained from $\delta$-independent polarimetric parameters $CA_{CD}$ (Eq. 13) and *L* (Eq. 12). Fig. 2 a, f shows $CA_{CD}$ values distributed uniformly in the longitudinal cut, while the distribution is broader in the oblique cut, with larger absolute $CA_{CD}$ values. In comparison, the values of *L* are only slightly larger in the oblique cut than in the longitudinal cut (Fig. 2 b, g). This is due to *L* approaching asymptotic values for collagen (when $R\approx 1.7$). Therefore, only $CA_{CD}$ can be used as a proxy to estimate the *R* ratio in collagen. Note that the relation between *R* and $CA_{CD}$ is not linear.

The *R* ratio can be calculated using Eqs. (20) and (21). The $CA_{CD}$ equation (Eq. (20)) requires assuming that $C^2 = 0$, while the combined $CA_{CD} \& L$ equation (Eq. (21)) is independent of *C*. This makes calculations with Eq. (21) more accurate when *C* is large. Fig. 2 shows *R* ratio maps calculated using Eq. (20) (panels c, h) and Eq. (21) (panels d, i), and the PIPO fit results (panels e, j). The maps obtained with all three methods are comparable, although *R* values calculated with DSP are slightly smaller (Fig. 2) having median and IQR values: $1.53\pm 0.25$ (Eq. (20), $CA_{CD}$ eq.), $1.53\pm 0.24$ (Eq. (21), $CA_{CD}$ & *L* eq.) and $1.50\pm 0.13$ (PIPO fit) for the longitudinal cut sample, $1.93\pm 0.49$ (Eq. (20), $CA_{CD}$ eq.), $1.93\pm 0.49$ (Eq. (21), $CA_{CD}$ & *L* eq.) and $2.14\pm 0.68$ (PIPO fit) for the oblique cut sample. Note that the numerical values of *R* are provided for the H&E stained tendon sample. The unstained tendon has slightly lower *R* ratio. Simulations reveal an underestimation of the *R* ratio obtained with DSP due to sample birefringence ($\sim$2 % for the longitudinal fibers, $\sim$7 % for the oblique cut fibers). Also, in the longitudinal cut, the *R* distribution obtained with PIPO fitting is slightly narrower (compare Fig. 2 c-e). The *R* ratio is higher in the oblique cut due to the dependence on out-of-plane tilt angle $\alpha$ (Eq. (3)). The filtering for pure polarization states has small effect on the *R* ratio (see Supplementary Material G). The highest difference is observed for the oblique cut, however, it does not exceed 0.1. In summary, $CA_{CD}$ (Eq. (20)) and $CA_{CD} \& L$ (Eq. (21)) can be used for quick calculation of *R* maps. Note that the *L* parameter alone can also be used for calculating *R* in the low-value range (Eq. (12)), e.g., for myofibrils ($R\approx 0.5$).

#### *C* ratio magnitude and phase difference $\Delta$

The C ratio is complex-valued, therefore, it can be expressed as the magnitude *C* and the phase difference $\Delta$ between the achiral and chiral nonlinear susceptibility tensor components. *T* and *H* polarimetric parameters measured with linear polarization states are proportional to $C\cos {\Delta }$ (Eqs. (14), (15)), while $SHG_{CD}$ measured with circular incident polarizations and *W* measured with linear incident and circular outgoing polarizations scale as $C\sin {\Delta }$ (Eqs. (17), (16)). The maps of *T*, *H*, $SHG_{CD}$ and *W* have relatively narrow value distributions centered around 0 due to $\sin {\alpha }\approx 0$ for in-plane oriented fibers (Fig. 3 a-d). A slight shift of the distribution to the negative values for *T*, *H*, *W* and to the positive values for $SHG_{CD}$ could be indicative of birefringence effects. The oblique cut fibers have a broader value distribution and have blue and red regions reflecting the polarity of collagen and influence of $\Delta$ (Fig. 3 e-h). *T* and *H* maps are similar, while $SHG_{CD}$ and *W* parameters have different value distributions and also are different from *T* and *H*. It has been shown that positive and negative $SHG_{CD}$ values (Eq. (16)) depend on the collagen polarity and also on the relative phase differences between the susceptibility components^23,26,27,31,32^. On the other hand, the sign of *T* and *H* is influenced only by the collagen polarity, since $\cos {\Delta }$ is an even function and its sign does not change in the [$-\frac{\pi }{2};\frac{\pi }{2}$] range. Note that *H* maps provide a direct measure of $\sigma$, which is used to calculate the fiber orientation angle $\delta$, and appears in many equations related to the linear polarization states. The $\sigma$ parameter reflects the organization of chiral fibers in a voxel. The value distributions of *T*, *H*, $SHG_{CD}$ and *W* parameters calculated without filtering for $s_0$ are similar to the filtered data (Supplementary Material G), showing DSP applicability for obtaining *C* and $\Delta$ in low scattering samples.

**Fig. 4:**
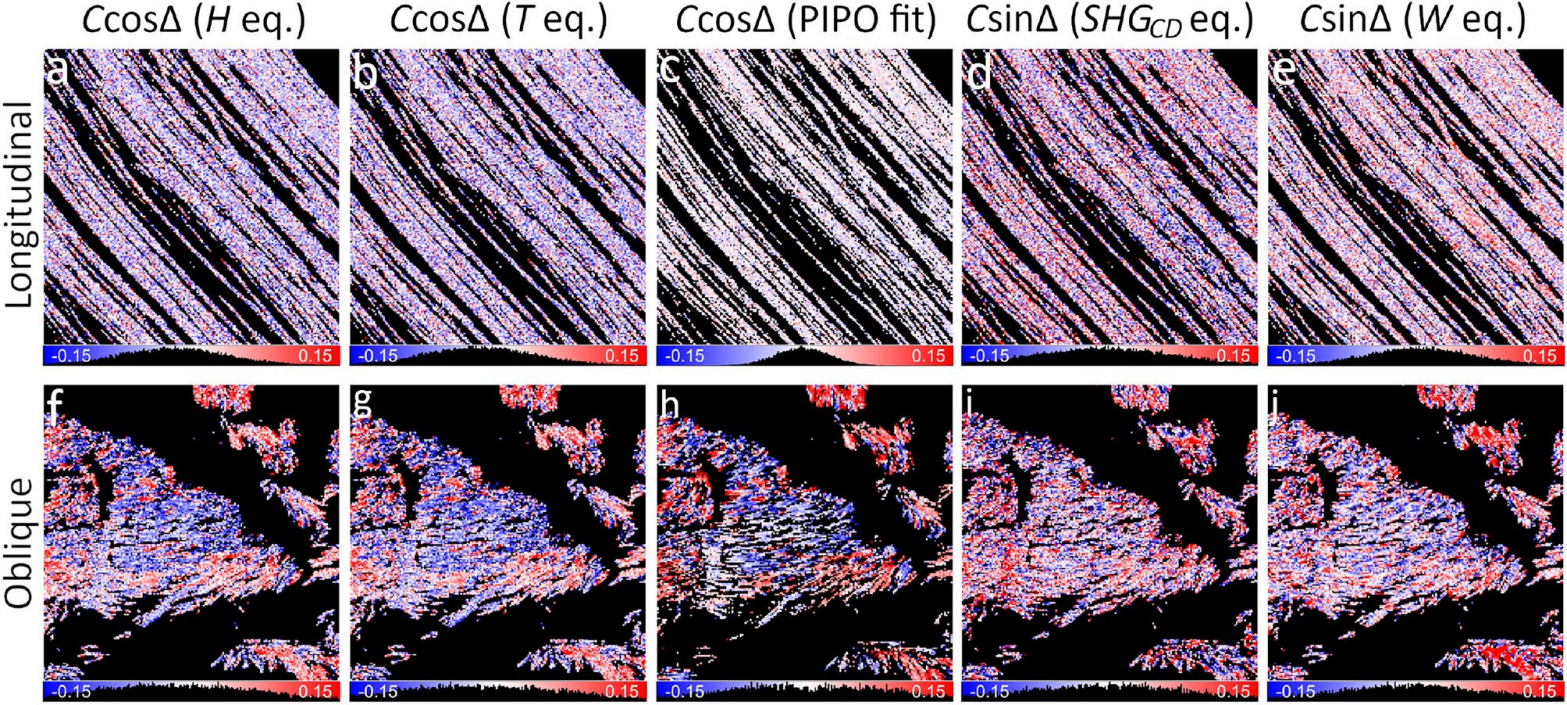
Maps of $C\cos {\Delta }$ (**a-c**, **f-h**) and $C\sin {\Delta }$ (**d**, **e** and **i**, **j**) deduced for the longitudinal (**a**-**e**) and oblique (**f**-**j**) cut of RTT. The $C\cos {\Delta }$ maps are calculated from the *H* parameter (Eq. (23)) (**a**, **f**), *T* parameter (Eq. (22)) (**b**, **g**) and by PIPO fitting (**c**, **h**). The $C\sin {\Delta }$ maps are calculated from $SHG_{CD}$ with Eq. (24) (**d**, **i**) and from *W* with Eq. (25) (**e**, **j**). The polarimetric parameter maps of Fig. 3 are used for the calculations. Parameter histograms are shown under the corresponding maps. The color lookup bars are combined with the histograms.

**Fig. 5:**
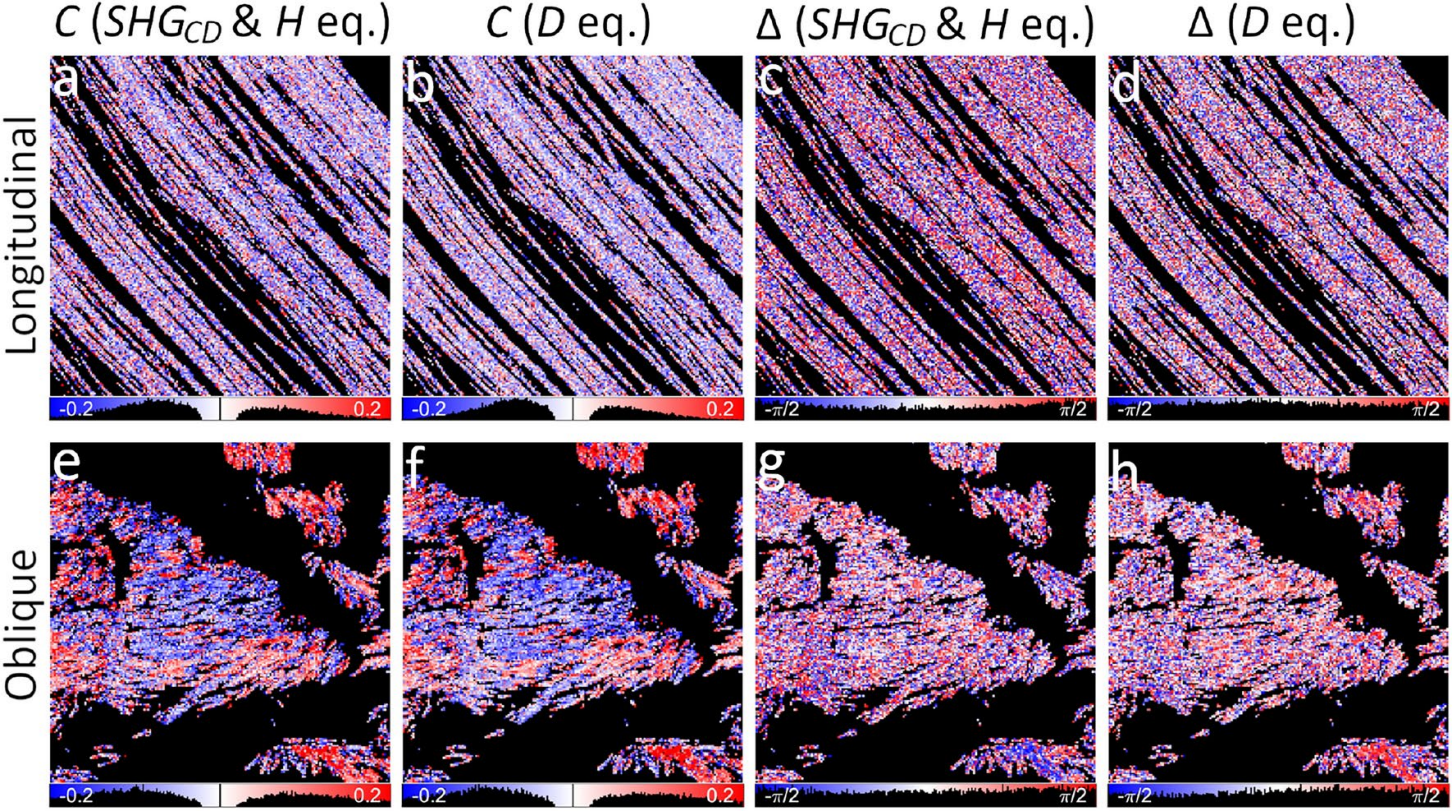
Magnitude *C* (**a**, **b**, **e**, **f**) and phase $\Delta$ (**c**, **d**, **g**, **h**) of the C ratio for the longitudinal (**a**-**d**) and oblique (**e**-**h**) cut of RTT. The *C* magnitude maps are calculated from Eqs. (23) and (24) (**a**, **e**), and from Eqs. (22) and (25) (**b**, **f**). The phase values are calculated using Eq. (26) (**c**, **g**) and Eq. (27) (**d**, **h**), and the pixels are made grey in **c**, **d**, **g**, **h** if the corresponding pixels have small absolute $C\cos {\Delta }$ and $C\sin {\Delta }$ values below 0.02 threshold. The same scan areas are used as in previous figures. Parameter histograms are shown under the corresponding maps. The color lookup bars are combined with the histograms.

The maps of $C\cos {\Delta }$ are calculated from *T* and *H* parameters and compared with $C\cos {\Delta }$ obtained from the PIPO fits (Fig. 4 a-c, f-h). The $C\cos \Delta$ maps are very similar for all three methods: $-0.02\pm 0.08$ (Eq. (23), $C\cos {\Delta }$ (*H* eq.)), $-0.02\pm 0.08$ (Eq. (22), $C\cos {\Delta }$ (*T* eq.)) and $-0.002\pm 0.04$ ($C\cos {\Delta }$ (PIPO fit)) for the longitudinal sample, $-0.02\pm 0.13$ (Eq. (23), $C\cos {\Delta }$ (*H* eq.)), $-0.02\pm 0.13$ (Eq. (22), $C\cos {\Delta }$ (*T* eq.)) and $-0.001\pm 0.13$ ($C\cos {\Delta }$ (PIPO fit)) for the oblique sample. The distribution from the PIPO fit is slightly narrower for the longitudinal cut (Fig. 4 c) due to fitting. On the other hand, $C\sin {\Delta }$ maps are obtained from $SHG_{CD}$ and *W* parameters (Fig. 4 d, i and e, j, respectively). As expected, $C\sin {\Delta }$ distributions are centered around 0 and are narrower in the longitudinal cut compared to the oblique cut (Fig. 4 d, e) due to *C* dependence on $\sin {\alpha }$ (Eq. (4)). The distributions of $C\sin {\Delta }$ calculated with the *W* parameter are narrower than ones calculated with the $SHG_{CD}$ (compare Fig. 4 d with e and i with j): $-0.004\pm 0.11$ (Eq. (24), $C\sin {\Delta }$ ($SHG_{CD}$ eq.)), $0.004\pm 0.08$ (Eq. (25), $C\sin {\Delta }$ (*W* eq.)) for the longitudinal sample, $-0.003\pm 0.14$ (Eq. (24), $C\sin {\Delta }$ ($SHG_{CD}$ eq.)), $0.001\pm 0.13$ (Eq. (25), $C\sin {\Delta }$ (*W* eq.)) for the oblique sample. Note that some corresponding regions have opposite signs in $C\sin {\Delta }$ maps calculated from $SHG_{CD}$ and *W*. This happens due to birefringence affecting differently the double-Stokes vector of the fundamental light and Stokes vector of the SHG, and may also be influenced by quadrupolar and magnetic interactions^27,33^, which are not accounted for by the CCS model. The $C\sin {\Delta }$ images have different spatial distributions of positive and negative values compared to $C\cos {\Delta }$ maps, pointing to the influence of the $\Delta$ function. The $\Delta$ can assume positive or negative values depending on the fiber configuration in the focal volume.

Having both $C\sin {\Delta }$ and $C\cos {\Delta }$ allows to separate *C* and $\Delta$. The $\Delta$ maps (Fig. 5) are calculated using two alternative equations, Eqs. (26) and (27). The former requires to determine the *R* ratio and to use $SHG_{CD}$ and *H* to calculate $\tan {\Delta }$. The latter equation uses the *D* parameter, which is the ratio between *W* and *T* parameters, and allows direct measurements of $\tan {\Delta }$. Obtaining $\tan \Delta$ with *D* parameter (Eq. (27)) is expected to give more accurate results, as it does not require assuming that $C^2$ is small. The $\Delta$ can be determined if at least one of $C\sin {\Delta }$ or $C\cos {\Delta }$ is not close to 0. In this work, 0.02 was selected as the threshold value, and pixels having both $C\sin {\Delta }$ and $C\cos {\Delta }$ less than the threshold were removed from the $\Delta$ maps shown in Fig. 5 c, d, g, h. Eq. (26) uses $SHG_{CD}$, measured with incoming circular polarizations, which results in relatively more pixels having $\Delta$ values near $\pm \pi /2$ (Fig. 5 c, g). In contrast, Eq. (27) utilizes outgoing circular polarizations via the *W* parameter and gives $\Delta$ values distributed closer to 0 (Fig. 5 d, h). The origin of $\Delta$ in collagen fibers requires further investigation^27^.

The *C* ratio magnitude can be determined from $C\cos {\Delta }$ and $C\sin {\Delta }$ equations by $\sqrt{(C\cos {\Delta })^2+(C\sin {\Delta })^2}$ and using the sign from $C\cos {\Delta }$. *C* maps calculated with Eqs. (23), (24) and Eqs. (22), (25) are shown in Fig. 5 (a, e) and (b, f), respectively. Both methods result in similar *C* maps. In the longitudinal cut, *C* has a relatively narrow distribution centered around 0 due to $\sin {\alpha }$ dependence (Fig. 5 a, b). The oblique cut sample shows characteristic collagen fiber polarity, with fibers in red and blue regions tilted out of the image plane in opposite directions (Fig. 5 e, f).

In summary, collagen polarity is reflected in *C* maps. Polarity can also be studied with directly measurable *H* and *T* parameters. Retardance $\Delta$ can be directly measured using the *D* parameter (Eq. (27)) or calculated from $SHG_{CD}$ and *H* (Eq. (26)). The equations give different $\Delta$ distributions, indicating that incident circular polarizations give rise to a different response compared to the linear incident and circular outgoing polarizations. Sample birefringence may cause these differences, particularly for in-image-plane oriented fibers measured with a combination of linear and circular polarizations. Quadrupolar and magnetic interactions may also play a role, in addition to the dipolar interactions^27^.

## Conclusions

The DSP method is validated with RTT sectioned at different orientations. The CCS model adequately describes the polarimetry measurements for the longitudinal and oblique cut RTT. The following ultrastructural parameters are obtained with DSP measurements that describe chiral fiber organization in a voxel:

1. The fiber orientation $\delta$ is deduced from the *A* parameter by using linear incident polarizations, or from the *B* parameter by using circular incident and linear outgoing polarizations. Note that the $\sigma$ parameter is used for the calculations and the signs of $SHG_{LD}$ and $SHG_{45}$ are used for extending $\delta$ values to the full orientation range.

2. The *R* ratio is obtained from the $CA_{CD}$ parameter by using circular polarization states. Alternatively, the *R* is obtained from the combination of $CA_{CD}$ and *L* parameters, which is applicable for large *C* values. Note that the *L* parameter employs linear incident and outgoing polarizations, therefore, both linear and circular polarizations are used for determining *R* by the latter method.

3. The ratio between chiral and achiral susceptibility component ratios, $\sigma$, is directly measured using the *H* parameter, which combines linear incident and outgoing polarizations. The $\sigma$ is used to correct for the deviation from the actual collagen orientation $\delta$ due to fiber chirality. Collagen polarity can be determined with the *H* parameter, and also with the *T* parameter.

4. The phase difference between the achiral and chiral susceptibility components, $\Delta$, is directly measured with the *D* parameter, which is the ratio of *W* and *T* parameters, and is valid when $C \ne 0$. The *D* parameter is obtained by using linear incident polarization states and measuring the whole outgoing SHG Stokes vector. Alternatively, the $\Delta$ can be obtained from the $SHG_{CD}$ and *T* measurements, but this method requires knowing the *R* ratio. Note that both $\Delta$ calculation methods give different results in some sample regions due to birefringence effects, and may also be influenced by quadrupolar and magnetic effects and therefore require further experimental investigations.

5. The *C* ratio magnitude is obtained from $C\cos {\Delta }$ and $C\sin {\Delta }$, which can be calculated using *T*, *H*, $SHG_{CD}$ and *W* parameters. The *C* ratio can be used to investigate the collagen polarity.

Recommendations are provided on the use of an optimal number of polarimetric measurements for the structural characterization of the samples:

1. Comprehensive information can be obtained about the sample by using incident circular polarizations and measuring the whole outgoing Stokes vector. The fiber orientation $\delta$ can be obtained from the *B* parameter, the *R* ratio from $CA_{CD}$, and the $C\sin {\Delta }$ from $SHG_{CD}$.

2. The use of incident linear polarization states and measurement of the whole outgoing Stokes vector provides the fiber orientation angle $\delta$ (*A* parameter) and allows separation of the *C* magnitude and phase $\Delta$ (*D* parameter). The *R* ratio can also be calculated by using the linear polarizations (*L* parameter). However, *L* reaches asymptotic values for collagen, resulting in low *R* determination accuracy. Nevertheless, the *L* can be used for the determination of low *R* ratios i.e. in muscle fibers with $R\approx 0.5$.

DSP polarimetric parameters can find applications in large-area imaging. Furthermore, the parameters requiring few polarimetric states (e.g. *R* ratio and $SHG_{CD}$) can be used for fast imaging with single-shot and double-shot measurements^26^. DSP is well suited for the structural characterization of collagen in whole slide SHG polarimetric imaging for histopathology investigations.

## Methods

### Biological samples

Albino Wistar female rats (10–11 weeks old) were acquired from the State Scientific Research Institute of the Innovative Medical Center (Vilnius, Lithuania). Rats were maintained at a constant temperature ($22^{\circ }$ C$\pm 1^{\circ }$ C), relative humidity 55% ± 10%, and a 12 h light/dark cycle. Animals were acclimatized for 7 days before the experiment. The animals were provided with autoclaved rodent chow (Diet 4RF25; Mucedola, Milan, Italy) and purified water ad libitum. One animal was sacrificed by cervical dislocation and the tail tendon samples were excised postmortem. The RTT samples were sectioned longitudinally and obliquely with a cryostat microtome to 10 $\upmu$m thick slices. The sections were stained using a standard hematoxylin and eosin (H&E) staining procedure with Mayer’s hematoxylin and Eosin Y (Leica ST5020 stainer). All animal procedures were carried out in accordance with the national and European regulations and were approved by the Lithuanian Animal Care and Use Committee of the State Food and Veterinary Service (Vilnius, Lithuania, approval number G2-156).

### Polarimetric measurements

Polarimetric SHG measurements were performed with a home-built laser scanning microscope. A femtosecond laser oscillator emitting at 1030 nm (FLINT, Light Conversion) was used for imaging. A pair of galvanometric mirrors (ScannerMAX, Pangolin Laser Systems) were used to scan the beam over the sample. Samples were imaged using a 20$\times$ 0.75 NA excitation objective (Plan Apo Lambda, Nikon) and a 0.4 NA singlet collection lens. SHG signal was detected with a photomultiplier tube (PMT) operating in photon counting mode (H10682-210, Hamamatsu). A 750 nm short-pass (Thorlabs) and a 515 nm bandpass (Edmund Optics) filter were inserted before the PMT to block the fundamental laser beam and collect the SHG light, respectively. Image acquisition was performed using custom software written in LabVIEW. Imaging was performed on $100 \times 100$ $\upmu$m sample areas with $200 \times 200$ pixel resolution. Laser power was 2 mW at the sample. Pixel dwell time was 25 $\upmu$s for the longitudinally cut sample, and 75 $\upmu$s for the obliquely cut sample due to the decrease in SHG intensity with increasing out-of-plane fibril tilt angle.

For polarimetric measurements, the microscope was equipped with a polarization state generator and analyzer, each consisting of a linear polarizer, a half-wave plate, and a quarter-wave plate (Fig. 1 a). The PSG was placed before the excitation objective, while the PSA was inserted before the PMT. The components of PSG and PSA were optimized for 1030 nm and 515 nm wavelengths, respectively.

To obtain the DSP polarimetric parameters, six incident polarizations were set using the PSG: HLP, VLP, 45, −45, RCP, and LCP. For every PSG polarization, six polarizations (HLP, VLP, 45, −45, RCP, and LCP) of the outgoing SHG signal were probed with the PSA (36 PSG/PSA combinations in total). Stokes vector components of the SHG signal were calculated for each of the six incident polarizations. These Stokes vector components were then used for DSP calculations.

For PIPO polarimetry, eight linear PSG states from 0$^{\circ }$ to 180$^{\circ }$ in steps of 22.5$^{\circ }$ were used for excitation. For each PSG state, linear PSA states from 0$^{\circ }$ to 180$^{\circ }$ in steps of 22.5$^{\circ }$ were used to probe the outgoing SHG signal (64 PSG/PSA combinations).

### Consistency checks and data filtering

Degree of polarization filtering was performed on the acquired data^34^. The $s_0$ Stokes components were calculated from the measured $s_1$, $s_2$ and $s_3$ components as $s_0^{2} = s_1^{2} + s_2^{2} + s_3^{2}$, since DSP is applicable for the pure polarization states. Polarization specific filtering was also performed according to $s_0^{RCP}-s_0^{LCP}=s_3^{RCP}+s_3^{LCP}$, and pixels appearing further away from the theoretical curve $s_0^{RCP}-s_0^{LCP}$ vs $s_3^{RCP}+s_3^{LCP}$ were excluded from the analysis.

### Calculation of DSP polarimetric and ultrastructural parameters

Stokes matrices were calculated from the measured intensity images for each pixel. Pixels having an average $s_0$ value lower than the intensity threshold (10 counts in this case) were discarded from further calculations. The maps of DSP polarimetric parameters were calculated by performing algebraic operations on the filtered intensity images of corresponding polarization states using equations outlined in the Theoretical considerations. The ultrastructural parameters (*R* ratio, *C* ratio magnitude, phase difference $\Delta$, and in-image-plane orientation angle $\delta$) were calculated using the corresponding equations of the theory section. The calculations were performed with custom MATLAB software.

PIPO fitting was performed with custom MATLAB software as described elsewhere^35^. The *R* ratio, $C\cos {\Delta }$, and fiber in-image-plane orientation angle $\delta$ were obtained from the fit.

### Simulation of polarimetric data

Simulations of the polarimetric data were performed using custom NLPS software written in MATLAB^34^. The software allows the simulation of nonlinear susceptibility tensors with specified parameters. In this work, we modeled collagen fibers with $C_6$ symmetry and CCS assumption. The nonlinear susceptibility tensor was specified to be complex. The $\chi _{zzz}$ component was set to 1.6 and the $\chi _{xyz}$ component to 0.2 + 0.2i. It is implicitly assumed that $\chi _{zxx}$ and $\chi _{xxz}$ components are equal to 1, therefore, $\chi _{zzz}$ and $\chi _{xyz}$ values correspond to *R* and *C* ratio values. The orientation angles $\alpha$ and $\delta$ were varied in the range [$-\frac{\pi }{2};\frac{\pi }{2}$]. The sample birefringence can be set as the phase delay, $\varphi$, value for both the fundamental and SHG wavelengths. The delay for the path length was calculated according to the equation:

28 $$\begin{aligned} \varphi = \frac{2\pi }{\lambda }\Delta nl, \end{aligned}$$

where $\lambda$ is 1030 nm or 515 nm for fundamental and SHG wavelengths, respectively. The $\Delta n$ was assumed to be equal to 0.002 for both wavelengths^36^. As the samples were 10 $\upmu$m thick, the path length *l* was set to be 5 $\upmu$m.

## Supplementary Information

Supplementary Information.

## Data Availability

The data supporting the findings of this study can be found in the published article. Should any raw image files be needed in another format they are available from V.B. upon reasonable request.

## Code availability

The NLPS software used for data simulations can be found at TSpace, University of Toronto: https://utoronto.scholaris.ca/bitstreams/184da5c1-72ca-4d9c-9187-deca5c0a4b82/download.

## References

[1] Roth, S. & Freund, I. Second harmonic generation in collagen. *Journal of Chemical Physics* **70**, 1637–1643. 10.1063/1.437677 (1979).

[2] Both, M., Vogel, M., Fink, R. & Uttenweiler, D. Second harmonic generation imaging in muscle fibers. In *Confocal, Multiphoton, and Nonlinear Microscopic Imaging*, 5139_112, 10.1364/ECBO.2003.5139_112 (2003).

[3] Mizutani, G. et al. Detection of starch granules in a living plant by optical second harmonic microscopy. *Journal of Luminescence* **87–89**, 824–826. 10.1016/S0022-2313(99)00428-7 (2000).

[4] Carriles, R. et al. Invited Review Article: Imaging techniques for harmonic and multiphoton absorption fluorescence microscopy. *Review of Scientific Instruments* **80**, 081101. 10.1063/1.3184828 (2009). 19725639 10.1063/1.3184828 PMC2736611

[5] Tuer, A. E. et al. Hierarchical model of fibrillar collagen organization for interpreting the second-order susceptibility tensors in biological tissue. *Biophysical Journal* **103**, 2093–2105. 10.1016/j.bpj.2012.10.019 (2012). 23200043 10.1016/j.bpj.2012.10.019 PMC3512050

[6] Plotnikov, S. V., Millard, A. C., Campagnola, P. J. & Mohler, W. A. Characterization of the myosin-based source for second-harmonic generation from muscle sarcomeres. *Biophysical Journal* **90**, 693–703. 10.1529/biophysj.105.071555 (2006). 16258040 10.1529/biophysj.105.071555 PMC1367074

[7] Samim, M., Krouglov, S. & Barzda, V. Double stokes mueller polarimetry of second-harmonic generation in ordered molecular structures. *J. Opt. Soc. Am. B* **32**, 451–461. 10.1364/JOSAB.32.000451 (2015).

[8] Alizadeh, M. et al. Identifying crossing collagen fibers in human corneal tissues using pSHG images. *Biomed. Opt. Express* **10**, 3875–3888. 10.1364/BOE.10.003875 (2019). 31452981 10.1364/BOE.10.003875 PMC6701537

[9] Chu, S.-W. et al. Studies of (2)/(3) tensors in submicron-scaled bio-tissues by polarization harmonics optical microscopy. *Biophysical Journal* **86**, 3914–3922. 10.1529/biophysj.103.034595 (2004). 10.1529/biophysj.103.034595 PMC1304293 15189888

[10] Psilodimitrakopoulos, S. et al. In vivo, pixel-resolution mapping of thick filaments’ orientation in nonfibrilar muscle using polarization-sensitive second harmonic generation microscopy. *Journal of Biomedical Optics* **14**, 014001. 10.1117/1.3059627 (2009). 19256689 10.1117/1.3059627

[11] Amat-Roldan, I., Psilodimitrakopoulos, S., Loza-Alvarez, P. & Artigas, D. Fast image analysis in polarization SHG microscopy. *Opt. Express* **18**, 17209–17219. 10.1364/OE.18.017209 (2010). 20721110 10.1364/OE.18.017209

[12] Golaraei, A. et al. Collagen chirality and three-dimensional orientation studied with polarimetric second-harmonic generation microscopy. *Journal of Biophotonics* **12**, e201800241. 10.1002/jbio.201800241 (2019). 30288949 10.1002/jbio.201800241

[13] Golaraei, A. et al. Characterization of collagen in non-small cell lung carcinoma with second harmonic polarization microscopy. *Biomed. Opt. Express* **5**, 3562–3567. 10.1364/BOE.5.003562 (2014). 25360372 10.1364/BOE.5.003562 PMC4206324

[14] Tokarz, D. *et al.* Tumor tissue characterization using polarization-sensitive second harmonic generation microscopy. In D.D.S., C. K., M.D., K. S., Tromberg, B. J. & Bagnato, V. S. (eds.) *Biophotonics South America*, vol. 9531, 95310C, 10.1117/12.2180969 (2015).

[15] Tokarz, D. et al. Characterization of pathological thyroid tissue using polarization-sensitive second harmonic generation microscopy. *Laboratory Investigation* **100**, 1280–1287. 10.1038/s41374-020-0475-7 (2020). 32737408 10.1038/s41374-020-0475-7

[16] Tuer, A. E. et al. Nonlinear optical properties of type i collagen fibers studied by polarization dependent second harmonic generation microscopy. *The Journal of Physical Chemistry B* **115**, 12759–12769. 10.1021/jp206308k (2011). 21970315 10.1021/jp206308k

[17] Golaraei, A. et al. Complex susceptibilities and chiroptical effects of collagen measured with polarimetric second-harmonic generation microscopy. *Scientific Reports* **9**, 12488. 10.1038/s41598-019-48636-w (2019). 31462663 10.1038/s41598-019-48636-w PMC6713739

[18] Radaelli, F. et al. MAPPS: a novel phasor approach to second harmonic analysis for in vitro-in vivo investigation of collagen microstructure. *Scientific Reports* **7**, 17468. 10.1038/s41598-017-17726-y (2017). 10.1038/s41598-017-17726-y PMC5727101 29234132

[19] Alizadeh, M., Ghotbi, M., Loza-Alvarez, P. & Merino, D. Comparison of different polarization sensitive second harmonic generation imaging techniques. *Methods and Protocols* **2**, 49. 10.3390/mps2020049 (2019). 31181703 10.3390/mps2020049 PMC6632172

[20] Psilodimitrakopoulos, S., Loza-Alvarez, P. & Artigas, D. Fast monitoring of in-vivo conformational changes in myosin using single scan polarization-shg microscopy. *Biomed. Opt. Express* **5**, 4362–4373. 10.1364/BOE.5.004362 (2014). 25574444 10.1364/BOE.5.004362 PMC4285611

[21] Mazumder, N. et al. Polarization-resolved second harmonic generation microscopy with a four-channel stokes-polarimeter. *Opt. Express* **20**, 14090–14099. 10.1364/OE.20.014090 (2012). 22714473 10.1364/OE.20.014090

[22] Mazumder, N. et al. Stokes vector based polarization resolved second harmonic microscopy of starch granules. *Biomed. Opt. Express* **4**, 538–547. 10.1364/BOE.4.000538 (2013). 23577289 10.1364/BOE.4.000538 PMC3617716

[23] Petralli-Mallow, T., Wong, T. M., Byers, J. D., Yee, H. I. & Hicks, J. M. Circular dichroism spectroscopy at interfaces: a surface second harmonic generation study. *The Journal of Physical Chemistry* **97**, 1383–1388. 10.1021/j100109a022 (1993).

[24] Mirsanaye, K. et al. Machine learning-enabled cancer diagnostics with widefield polarimetric second-harmonic generation microscopy. *Scientific Reports* **12**, 10290. 10.1038/s41598-022-13623-1 (2022). 35717344 10.1038/s41598-022-13623-1 PMC9206659

[25] Uribe Castaño, L. et al. Wide-field stokes polarimetric microscopy for second harmonic generation imaging. *Journal of Biophotonics* **16**, e202200284. 10.1002/jbio.202200284 (2023). 36651498 10.1002/jbio.202200284

[26] Golaraei, A., Kontenis, L., Karunendiran, A., Stewart, B. A. & Barzda, V. Dual- and single-shot susceptibility ratio measurements with circular polarizations in second-harmonic generation microscopy. *Journal of Biophotonics* **13**, e201960167. 10.1002/jbio.201960167 (2020). 31975533 10.1002/jbio.201960167

[27] Schmeltz, M. et al. Circular dichroism second-harmonic generation microscopy probes the polarity distribution of collagen fibrils. *Optica* **7**, 1469–1476. 10.1364/OPTICA.399246] (2020).

[28] Chen, X., Raggio, C. & Campagnola, P. J. Second-harmonic generation circular dichroism studies of osteogenesis imperfecta. *Opt. Lett.* **37**, 3837–3839. 10.1364/OL.37.003837 (2012). 23041876 10.1364/ol.37.003837 PMC4337953

[29] Mirsanaye, K. et al. Unsupervised determination of lung tumor margin with widefield polarimetric second-harmonic generation microscopy. *Scientific Reports* **12**, 20713. 10.1038/s41598-022-24973-1 (2022). 36456811 10.1038/s41598-022-24973-1 PMC9715953

[30] Samim, M., Krouglov, S. & Barzda, V. Nonlinear stokes-mueller polarimetry. *Phys. Rev. A* **93**, 013847. 10.1103/PhysRevA.93.013847 (2016).

[31] Verbiest, T. et al. Nonlinear optical activity and biomolecular chirality. *Journal of the American Chemical Society* **116**, 9203–9205. 10.1021/ja00099a040 (1994).

[32] Harvey, M. et al. Histological staining alters circular dichroism shg measurements of collagen. *Opt. Lett.* **49**, 3705–3708. 10.1364/OL.523689 (2024). 38950247 10.1364/OL.523689

[33] Abramavicius, D., Krouglov, S. & Barzda, V. Second harmonic generation theory for a helical macromolecule with high sensitivity to structural disorder. *Phys. Chem. Chem. Phys.* **23**, 20201–20217. 10.1039/D1CP00694K (2021). 34473146 10.1039/d1cp00694k

[34] Kontenis, L. *Experimental Nonlinear Polarimetric Microscopy*. Ph.D. thesis, University of Toronto (2017).

[35] Golaraei, A. et al. Collagen chirality and three-dimensional orientation studied with polarimetric second-harmonic generation microscopy. *Journal of Biophotonics* **12**, e201800241. 10.1002/jbio.201800241 (2019). 30288949 10.1002/jbio.201800241

[36] Silva, D. F. T., Gomes, A. S. L., de Campos Vidal, B. & GRibeiro, M. S. Birefringence and second harmonic generation on tendon collagen following red linearly polarized laser irradiation. *Annals of Biomedical Engineering* **41**, 752–762, 10.1007/s10439-012-0720-3 (2013). 10.1007/s10439-012-0720-3 23247985

[37] Barzda, V., Kontenis, L., Maciulis, M. & Mazeika, V. Method for fast determination of ultrastructural parameters using polarimetric second harmonic generation microscopy (2023). European Patent Office, patent no. EP4290290.

[38] Lee, H. et al. Chiral imaging of collagen by second-harmonic generation circular dichroism. *Biomed. Opt. Express* **4**, 909–916. 10.1364/BOE.4.000909 (2013). 23761852 10.1364/BOE.4.000909 PMC3675869

