# Supplementary material for: Double Stokes polarimetric microscopy for chiral fibrillar aggregates
Source: Sci Rep. 2025 Feb 6;15:4464. doi: 10.1038/s41598-025-86893-0 (PMC11803116; doi:10.1038/s41598-025-86893-0)
Supplement: Supplementary file 1 — Supplementary Information. [file 41598_2025_86893_MOESM1_ESM.pdf]

Supplementary Material

Double Stokes Polarimetric Microscopy for Chiral Fibrillar Aggregates

Viktoras Mazeika[1,2], Kamdin Mirsanaye[3,4,5], Leonardo Uribe Castaño[3,4], Serguei Krouglov[3,4], Mehdi Alizadeh[2,3,4], Mykolas Maciulis[2], Lukas Kontenis[2,6], Vitalijus Karabanovas[2,7], and Virginijus Barzda[2,3,4,*]

[1]Institute of Biosciences, Life Sciences Center, Vilnius University, Vilnius, Lithuania
[2]Laser Research Centre, Faculty of Physics, Vilnius University, Vilnius, Lithuania
[3]Department of Chemical and Physical Sciences, University of Toronto Mississauga, Mississauga, ON, Canada
[4]Department of Physics, University of Toronto, Toronto, ON, Canada
[5]Wellman Center for Photomedicine and Center for Systems Biology, Massachusetts General Hospital and Harvard Medical School, Boston, MA, USA
[6]Light Conversion, Vilnius, Lithuania
[7]Biomedical Physics Laboratory, National Cancer Institute, Vilnius, Lithuania
[*]Corresponding author: virgis.barzda@utoronto.ca

A Double Stokes vector

Double Stokes vector formalism can be used to describe two electric fields of laser light. For the degenerate SHG case, the double Stokes vector is described by a 9-component vector as follows [1]:

$$S = \begin{bmatrix} S_1 \\ S_2 \\ S_3 \\ S_4 \\ S_5 \\ S_6 \\ S_7 \\ S_8 \\ S_9 \end{bmatrix} = \begin{bmatrix} \sqrt{1/6}(3s_0^2 - s_1^2) \\ \sqrt{1/12}(5s_1^2 - 3s_0^2) \\ s_0 s_1 \\ 1/2(s_2^2 - s_3^2) \\ -s_2(s_1 - s_0) \\ s_2(s_1 + s_0) \\ s_2 s_3 \\ s_3(s_1 - s_0) \\ s_3(s_1 + s_0) \end{bmatrix} \tag{S.1}$$

The double Stokes vector is expressed in terms of the first-order Stokes vector components s_0, s_1, s_2 and s_3 of a laser beam, analogous to the Eq. (1). The double Stokes vector is obtained via coherency matrix formalism using Gell-Mann matrices presented in Supplementary Material C [1].

B Relation between Mueller matrix and molecular susceptibilities

The double Stokes-Mueller formalism (Eq. (2)) enables the calculation of the double Mueller matrix M [1]. The double Mueller matrix elements are related to the second-order susceptibilities χ in the laboratory coordinate reference frame:

$$M_{\alpha N} = \frac{1}{2} Tr(\tau_\alpha \chi \lambda_N \chi^\dagger), \tag{S.2}$$

where τ_α and λ_N are the Pauli and Gell-Mann matrices, respectively, with index $\alpha = 0...3$, and index $N = 1...9$ (see τ_α and λ_N matrices in the Supplementary Material C). The susceptibilities are presented in the contracted notation $\chi_{IJK} = \chi_{IA}$, where the susceptibility tensor component index I corresponds to the laboratory coordinate system X and Z axes, and A is the contracted index representing XX, ZZ, and XZ or ZX [2]. Thus, χ is a 2×3 rectangular matrix. The superscript $\dagger$ denotes Hermitian conjugation.

The calculated laboratory frame nonlinear susceptibilities χ_{IJK} can be related to the molecular susceptibilities χ_{ijk} via tensor rotation [3]:

$$\chi_{IJK} = T_{Ii} T_{Jj} T_{Kk} \chi_{ijk}, \tag{S.3}$$

where T_{Nn} are the rotation matrices, and Einstein summation is assumed. Therefore, the SHG Stokes vector components (Eq. (1)) can be related to the molecular susceptibility tensor elements.

C Pauli and Gell-Mann matrices

The following Pauli (Eq. (S.4) and Gell-Mann (Eq. (S.5)) matrices are used for obtaining the expressions for the Stokes vector Eq. (1), the double Stokes vector (Eq. (S.1)), and the double Mueller matrix elements (Eq. (S.2)):

$$\tau_0 = \begin{pmatrix} 1 & 0 \\ 0 & 1 \end{pmatrix} \tau_1 = \begin{pmatrix} -1 & 0 \\ 0 & 1 \end{pmatrix} \tau_2 = \begin{pmatrix} 0 & 1 \\ 1 & 0 \end{pmatrix} \tau_3 = \begin{pmatrix} 0 & i \\ -i & 0 \end{pmatrix}; \tag{S.4}$$

$$\begin{gathered} \lambda_1 = \sqrt{\tfrac{2}{3}} \begin{pmatrix} 1 & 0 & 0 \\ 0 & 1 & 0 \\ 0 & 0 & 1 \end{pmatrix} \quad \lambda_2 = \sqrt{\tfrac{1}{3}} \begin{pmatrix} 1 & 0 & 0 \\ 0 & 1 & 0 \\ 0 & 0 & -2 \end{pmatrix} \quad \lambda_3 = \begin{pmatrix} 1 & 0 & 0 \\ 0 & -1 & 0 \\ 0 & 0 & 0 \end{pmatrix} \\ \lambda_4 = \begin{pmatrix} 0 & 1 & 0 \\ 1 & 0 & 0 \\ 0 & 0 & 0 \end{pmatrix} \quad \lambda_5 = \begin{pmatrix} 0 & 0 & 0 \\ 0 & 0 & 1 \\ 0 & 1 & 0 \end{pmatrix} \quad \lambda_6 = \begin{pmatrix} 0 & 0 & 1 \\ 0 & 0 & 0 \\ 1 & 0 & 0 \end{pmatrix} \\ \lambda_7 = \begin{pmatrix} 0 & -i & 0 \\ i & 0 & 0 \\ 0 & 0 & 0 \end{pmatrix} \quad \lambda_8 = \begin{pmatrix} 0 & 0 & 0 \\ 0 & 0 & -i \\ 0 & i & 0 \end{pmatrix} \quad \lambda_9 = \begin{pmatrix} 0 & 0 & -i \\ 0 & 0 & 0 \\ i & 0 & 0 \end{pmatrix} \end{gathered} \tag{S.5}$$

D Relations between the laboratory and the molecular frame nonlinear susceptibility tensor components

The laboratory reference frame nonlinear susceptibility tensor components can be expressed in terms of the molecular achiral R ratio (Eq. (3)), the molecular chiral C ratio magnitude and the phase difference Δ (Eq. (4)), and the in-plane orientation angle δ [4]:

$$\frac{\chi_{XXX}}{\chi'_{zxx}} = \sin\delta[(R-3)\sin^2\delta + 3] \tag{S.6}$$

$$\frac{\chi_{XXZ}}{\chi'_{zxx}} = \cos\delta[(R-3)\sin^2\delta + 1] - Ce^{i\Delta}\sin\delta \tag{S.7}$$

$$\frac{\chi_{XZZ}}{\chi'_{zxx}} = \sin\delta[(R-3)\cos^2\delta + 1] - 2Ce^{i\Delta}\cos\delta \tag{S.8}$$

$$\frac{\chi_{ZXX}}{\chi'_{zxx}} = \cos\delta[(R-3)\sin^2\delta + 1] + 2Ce^{i\Delta}\sin\delta \tag{S.9}$$

$$\frac{\chi_{ZXZ}}{\chi'_{zxx}} = \sin\delta[(R-3)\cos^2\delta + 1] + Ce^{i\Delta}\cos\delta \tag{S.10}$$

$$\frac{\chi_{ZZZ}}{\chi'_{zxx}} = \cos\delta[(R-3)\cos^2\delta + 3] \tag{S.11}$$

where $\chi'_{zxx} = \chi_{zxx}\cos\alpha$.

E Sums of Stokes vector components

The following are expressions of the sums of SHG Stokes vector components in terms of the molecular susceptibility ratios. The equations are obtained for specific incident polarization states that are described by the double Stokes vector (Eq. (S.1)). The Stokes vector is calculated using Stokes-Mueller formalism (Eqs. (2) and (S.2)), and the relations between laboratory and molecular frame susceptibilities for C_6 symmetry fibers (Eqs. (S.6), (S.7), (S.8), (S.9), (S.10), (S.11)).

E.1 0° and 90° incident linear polarizations

$$s_0^{HLP} + s_0^{VLP} = \frac{1}{4}\left((R-3)(R+1)\cos(4\,\delta)\; - 4\,(R-3)C\,\cos(\Delta)\,\sin(4\,\delta)\; + 3\,R^2 + 2\,R + 7 + 16\,C^2\right) \tag{S.12}$$

$$s_1^{HLP} + s_1^{VLP} = -\frac{1}{8}\left(\left(7R^2 + 6\,R - 1 - 32\,C^2\right)\,\cos(2\,\delta) + 16(R-1)\;C\,\cos(\Delta)\,\sin(2\,\delta) + (R-3)^2\cos(6\,\delta)\right) \tag{S.13}$$

$$s_2^{HLP} + s_2^{VLP} = \frac{1}{8}\left(-32\,R\,C\,\cos(\Delta)\,\cos(2\,\delta) + \left(5\,R^2 + 2\,R - 3\right)\,\sin(2\,\delta) + (R-3)^2\sin(6\,\delta)\right) \tag{S.14}$$

$$s_3^{HLP} + s_3^{VLP} = -(R-3)C\sin(\Delta)\cos(4\delta) - 3\,(R+1)C\sin(\Delta) \tag{S.15}$$

E.2 45° and -45° incident linear polarizations

$$s_0^{+45} + s_0^{-45} = \frac{1}{4}\left(-(R-3)(R+1)\,\cos(4\,\delta) + 4(R-3)C\,\cos(\Delta)\,\sin(4\,\delta) + 3\,R^2 + 2\,R + 7 + 16\,C^2\right) \tag{S.16}$$

$$s_1^{+45} + s_1^{-45} = -\frac{1}{8}\left(\left(5R^2 + 2\,R - 3\right)\,\cos(2\,\delta) + 32\,R\,C\,\cos(\Delta)\,\sin(2\,\delta) - (R-3)^2\cos(6\,\delta)\right) \tag{S.17}$$

$$s_2^{+45} + s_2^{-45} = \frac{1}{8}\left(-16\,(R-1)\,C\,\cos(\Delta)\,\cos(2\,\delta) + \left(7\,R^2 + 6\,R - 1 - 32\,C^2\right)\,\sin(2\,\delta) - (R-3)^2\sin(6\,\delta)\right) \tag{S.18}$$

$$s_3^{+45} + s_3^{-45} = (R-3)C\sin(\Delta)\cos(4\delta) - 3\,(R+1)C\sin(\Delta) \tag{S.19}$$

E.3 Left and right incident circular polarizations

$$s_0^{RCP} + s_0^{LCP} = \frac{1}{2}\left((R-1)^2 + 4 + 8\,C^2\right) \tag{S.20}$$

$$s_1^{RCP} + s_1^{LCP} = -\frac{1}{2}\left((R-3)(R+1)\cos(2\delta) + 4(R-3)C\cos(\Delta)\sin(2\delta)\right) \tag{S.21}$$

$$s_2^{RCP} + s_2^{LCP} = \frac{1}{2}\left((R-3)(R+1)\sin(2\delta) - 4(R-3)C\cos(\Delta)\cos(2\delta)\right) \tag{S.22}$$

$$s_3^{RCP} + s_3^{LCP} = -2(R+1)C\sin(\Delta) \tag{S.23}$$

F Differences of Stokes vector components

The differences of SHG Stokes vector components are calculated the same way as the sums described in the previous section. The ratios of the differences and sums of the Stokes vector components provide the expressions of DSP polarimetric parameters described in the article.

F.1 0° and 90° incident linear polarizations

$$s_0^{HLP} - s_0^{VLP} = 2\,(R-1)\,C\,\cos(\Delta)\,\sin(2\,\delta) - \left((R-1)\,(R+1) + 4\,C^2\right)\cos(2\,\delta) \tag{S.24}$$

$$s_1^{HLP} - s_1^{VLP} = \frac{1}{2}\left((R-3)\,(R+1)\,\cos(4\,\delta) + 2\,(R-3)\,C\,\cos(\Delta)\,\sin(4\,\delta) + (R+1)^2 - 8\,C^2\right) \tag{S.25}$$

$$s_2^{HLP} - s_2^{VLP} = \frac{1}{2}\left(2\,(R-3)\,C\,\cos(\Delta)\,\cos(4\,\delta) - (R-3)\,(R+1)\,\sin(4\,\delta) + 6\,(R+1)\,C\,\cos(\Delta)\right) \tag{S.26}$$

$$s_3^{HLP} - s_3^{VLP} = 4\,R\,C\,\sin(\Delta)\,\cos(2\,\delta) \tag{S.27}$$

F.2 45° and -45° incident linear polarizations

$$s_0^{+45} - s_0^{-45} = \left((R-1)\,(R+1) + 4\,C^2\right)\sin(2\,\delta) + 2\,(R-1)\,C\,\cos(\Delta)\,\cos(2\,\delta) \tag{S.28}$$

$$s_1^{+45} - s_1^{-45} = \frac{1}{2}\left(2\,(R-3)\,C\,\cos(\Delta)\,\cos(4\,\delta) - (R-3)\,(R+1)\,\sin(4\,\delta) - 6\,(R+1)\,C\,\cos(\Delta)\right) \tag{S.29}$$

$$s_2^{+45} - s_2^{-45} = \frac{1}{2}\left(-(R-3)\,(R+1)\,\cos(4\,\delta) - 2\,(R-3)\,C\,\cos(\Delta)\,\sin(4\,\delta) + (R+1)^2 - 8\,C^2\right) \tag{S.30}$$

$$s_3^{+45} - s_3^{-45} = -4\,R\,C\,\sin(\Delta)\,\sin(2\,\delta) \tag{S.31}$$

F.3 Left and right incident circular polarizations

$$s_0^{RCP} - s_0^{LCP} = -2\,(R+1)\,C\,\sin(\Delta) \tag{S.32}$$

$$s_1^{RCP} - s_1^{LCP} = 2\,(R-3)\,C\,\sin(\Delta)\,\cos(2\,\delta) \tag{S.33}$$

$$s_2^{RCP} - s_2^{LCP} = -2\,(R-3)\,C\,\sin(\Delta)\,\sin(2\,\delta) \tag{S.34}$$

$$s_3^{RCP} - s_3^{LCP} = 2\,(R-1) + 4\,C^2 \tag{S.35}$$

G Comparison of polarimetric and ultrastructural parameters calculated using directly measured and filtered pure s_0 states

The equations of the polarimetric and ultrastructural parameters are derived with the assumption of pure polarization states, i.e. $s_0^2 = s_1^2 + s_2^2 + s_3^2$. Filtering of the polarimetric data to obtain pure states necessitates measuring the entire Stokes vector. However, certain polarimetric parameters involve only a few Stokes vector components and can be obtained without measuring the entire SHG Stokes vector, provided that filtering for pure polarization states has a negligible effect due to the high degree of polarization (DOP) of the SHG. To investigate the influence

of data filtering for pure states, the polarimetric and ultrastructural parameters of the RTT samples are calculated by using directly measured s_0 Stokes component (without DOP filtering) and compared with values obtained by calculating s_0 from the measured s_1, s_2 and s_3 components (with DOP filtering). The measured s_0 values are subjected only to the physically realizable constraint $s_0^2 \geq s_1^2 + s_2^2 + s_3^2$.

Figs. S1 and S2 present the value distributions of polarimetric parameters used to calculate δ (i.e. A, B, SHG_{LD} and SHG_{45}) and δ orientation angle distributions of the longitudinal and oblique cut samples. The distributions of the polarimetric parameters and the calculated δ distributions are similar in both cases i.e. with and without DOP filtering. Therefore, for the presented cases, these parameters can be obtained using directly measured s_0, without DOP filtering.

Fig. S3 shows a comparison of the distributions of the R ratio as well as CA_{CD} and L parameters for the longitudinal and oblique cuts. The CA_{CD} and L values remain similar after DOP filtering for the longitudinal cut. The values of CA_{CD} and L slightly increase after DOP filtering for the oblique cut sample. Correspondingly, the filtering leads to slight increase of R ratio values by approximately 0.1.

Figs. S4 and S5 show the comparisons of C ratio and Δ as well as the parameters used to calculate them. The distributions of SHG_{CD}, H, T and W are similar with and without DOP filtering. Correspondingly, the C ratio amplitude and Δ phase distributions are similar with and without DOP filtering for both cuts.

The presented comparison shows that distributions for polarimetric and ultrastructural parameters calculated using directly measured and filtered pure polarization states are similar for both longitudinal and oblique cut RTT samples. The largest influence is observed for R parameter of the oblique cut. Nonetheless, the difference is small and the comparison of R between different samples is appropriate if the parameter is calculated with the same filtering method and has high DOP. The thin histology tissue sections investigated in this work have high degrees of polarization, which are about 0.9. Therefore, the polarimetric and ultrastructural parameters can be obtained by DSP method without filtering for pure states in tissues with high DOP values.

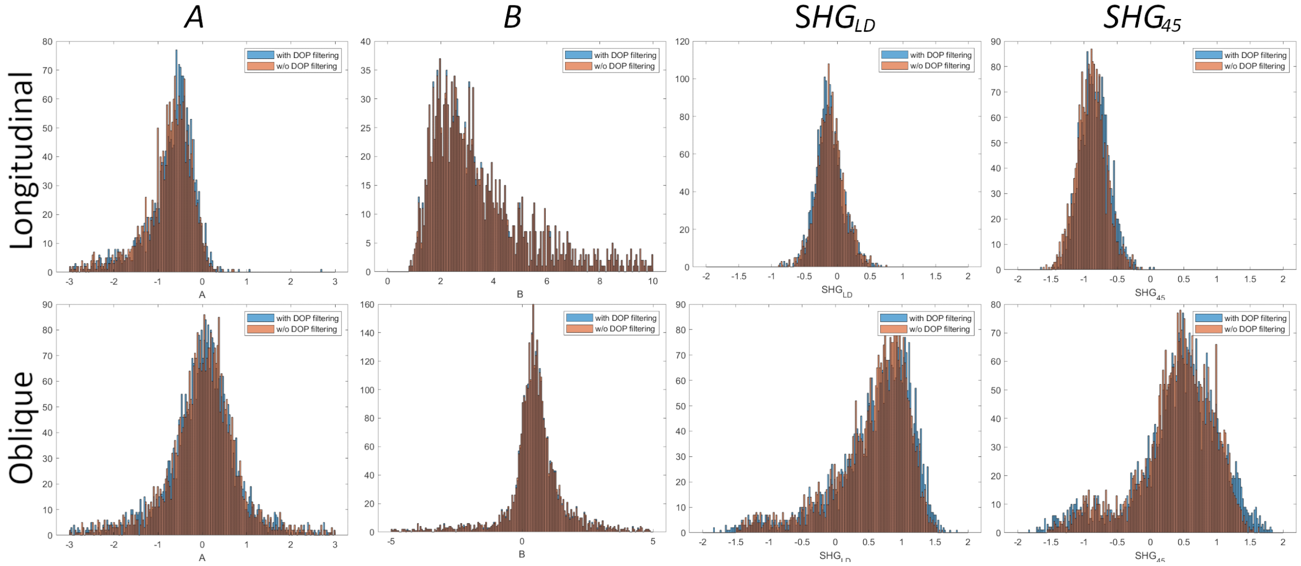

Fig. S1: Histograms of A, B, SHG_{LD} and SHG_{45} parameters calculated with and without DOP filtering for longitudinal and oblique cut rat tail tendon samples.

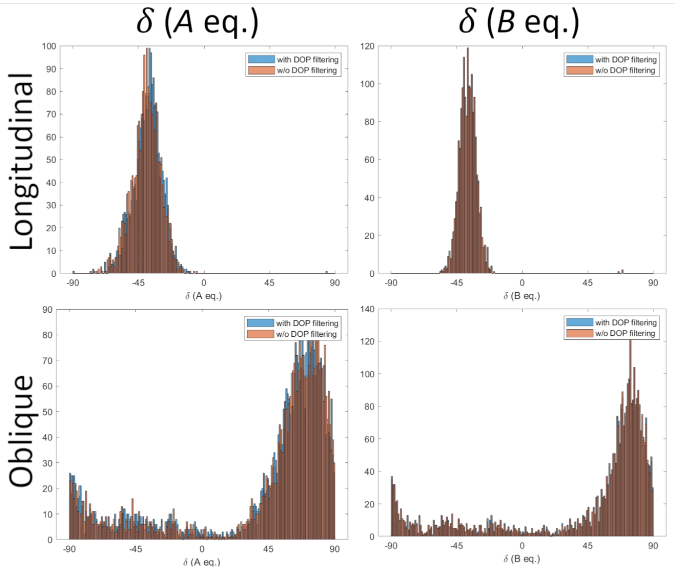

Fig. S2: Histograms of δ values calculated using A Eq. (18) (A eq.) and B Eq. (19) (B eq.) parameters with and without DOP filtering for longitudinal and oblique cut rat tail tendon samples.

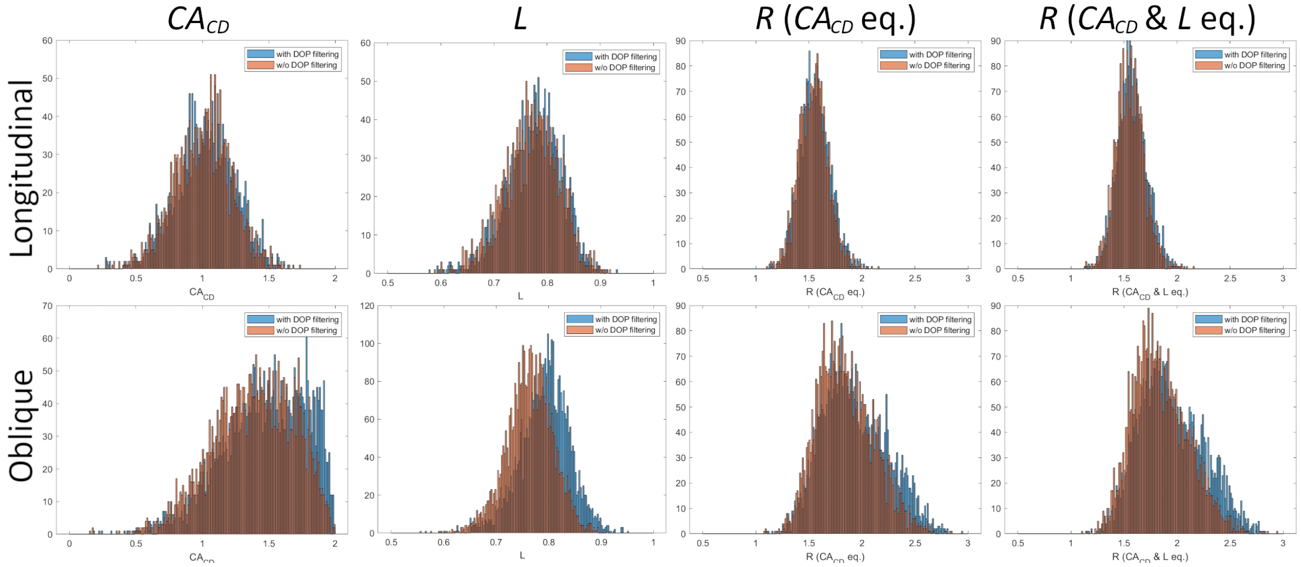

Fig. S3: Histograms of CA_{CD} and L polarimetric parameters, and R ratio calculated from CA_{CD} Eq. (20) (CA_{CD} eq.) and from combined CA_{CD} and L Eq. (21) (CA_{CD} & L eq.) obtained with and without DOP filtering for longitudinal and oblique cut rat tail tendon samples.

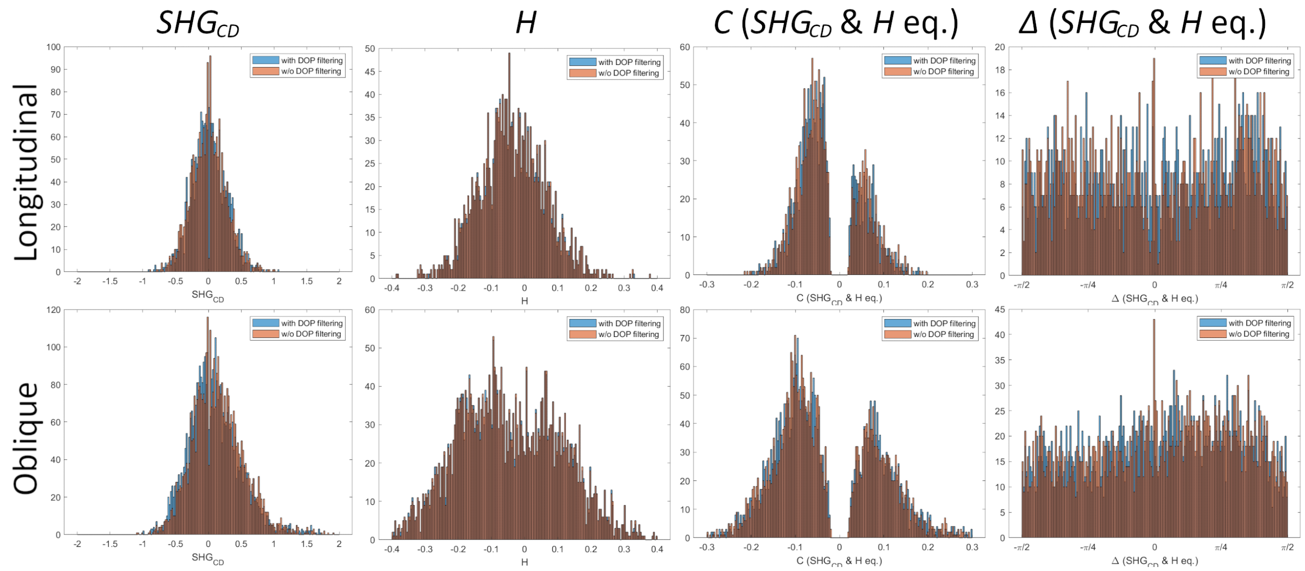

Fig. S4: Histograms of SHG_{CD}, H, as well as C ratio calculated with Eqs. (23) and (24) (SHG_{CD} & H eq.) and Δ calculated with Eq. (26) (SHG_{CD} & H eq.) for the cases with and without DOP filtering for longitudinal and oblique cut rat tail tendon samples.

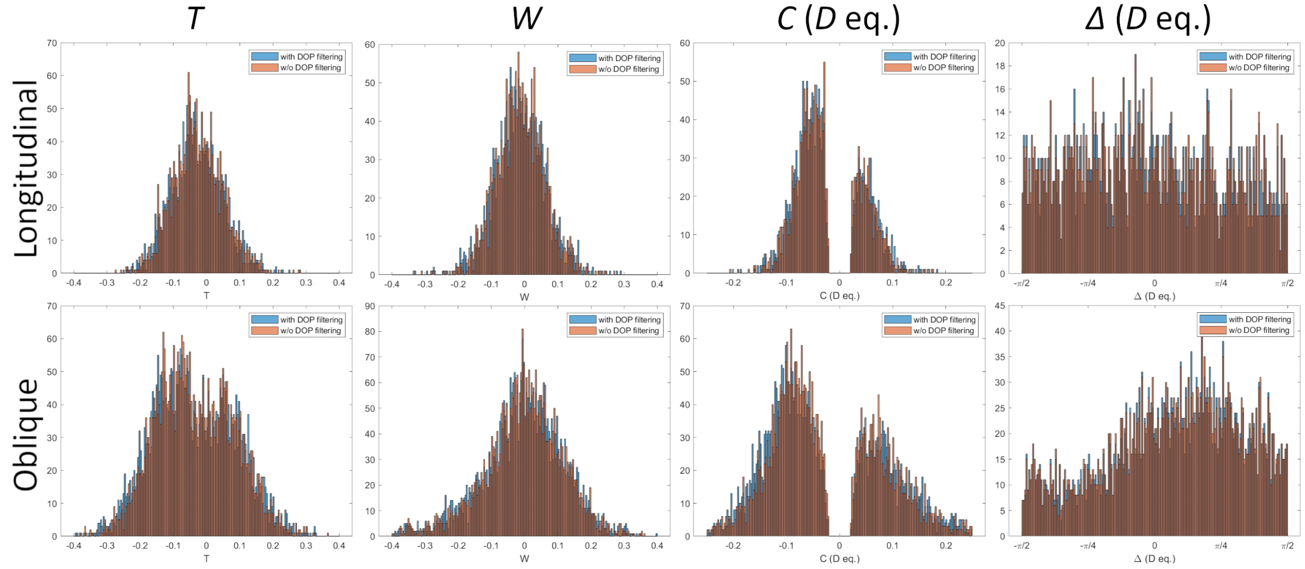

Fig. S5: Histograms of T, W, as well as C ratio calculated with Eqs. (22) and (25) (D eq.) and Δ calculated with Eq. (27) (D eq.) for the cases with and without DOP filtering for the longitudinal and oblique cut rat tail tendon samples.

References

[1] Samim, M., Krouglov, S. & Barzda, V. Double stokes mueller polarimetry of second-harmonic generation in ordered molecular structures. *J. Opt. Soc. Am. B* **32**, 451–461, 10.1364/JOSAB.32.000451 (2015).

[2] Boyd, R. W. *Nonlinear Optics, Fourth Edition* (Academic Press, Inc., 2020).

[3] Golaraei, A. *et al.* Collagen chirality and three-dimensional orientation studied with polarimetric second-harmonic generation microscopy. *J. Biophotonics* **12**, e201800241, 10.1002/jbio.201800241 (2019).

[4] Golaraei, A., Kontenis, L., Karunendiran, A., Stewart, B. A. & Barzda, V. Dual- and single-shot susceptibility ratio measurements with circular polarizations in second-harmonic generation microscopy. *J. Biophotonics* **13**, e201960167, 10.1002/jbio.201960167 (2020).
